# A novel kleptoplastidic symbiosis revealed in the marine centrohelid *Meringosphaera* with evidence of genetic integration

**DOI:** 10.1016/j.cub.2023.07.017

**Published:** 2023-08-01

**Authors:** Megan E.S. Sorensen, Vasily V. Zlatogursky, Ioana Onuţ-Brännström, Anne Walraven, Rachel A. Foster, Fabien Burki

**Affiliations:** 1 Department of Ecology, Environment and Plant Sciences, Stockholm University, 106 91 Stockholm, Sweden; 2 Institute of Microbial Cell Biology, Heinrich Heine University, 40225 Düsseldorf, Germany; 3 Department of Botany, University of British Columbia, V6T 1Z4 Vancouver, BC, Canada; 4 Department of Organismal Biology, Program in Systematic Biology, Uppsala University, 752 36 Uppsala, Sweden; 5 Department of Ecology and Genetics, Uppsala University, 752 36 Uppsala, Sweden; 6 Natural History Museum, University of Oslo, 0562 Oslo, Norway; 7 Science for Life Laboratory, Uppsala University, 752 37 Uppsala, Sweden

## Abstract

Plastid symbioses between heterotrophic hosts and algae are widespread and abundant in surface oceans. They are critically important both for extant ecological systems and for understanding the evolution of plastids. Kleptoplastidy, where the plastids of prey are temporarily retained and continuously re-acquired, provides opportunities to study the transitional states of plastid establishment. Here, we investigated the poorly studied marine centrohelid *Meringosphaera* and its previously unidentified symbionts using culture-independent methods from environmental samples. Investigations of the 18S rDNA from single-cell assembled genomes (SAGs) revealed uncharacterized genetic diversity within *Meringosphaera* that likely represents multiple species. We found that *Meringosphaera* harbors plastids of Dictyochophyceae origin (stramenopiles), for which we recovered six full plastid genomes and found evidence of two distinct subgroups that are congruent with host identity. Environmental monitoring by qPCR and catalyzed reporter deposition-fluorescence *in situ* hybridization (CARD-FISH) revealed seasonal dynamics of both host and plastid. In particular, we did not detect the plastids for 6 months of the year, which, combined with the lack of plastids in some SAGs, suggests that the plastids are temporary and the relationship is kleptoplastidic. Importantly, we found evidence of genetic integration of the kleptoplasts as we identified host-encoded plastid-associated genes, with evolutionary origins likely from the plastid source as well as from other alga sources. This is only the second case where host-encoded kleptoplast-targeted genes have been predicted in an ancestrally plastid-lacking group. Our results provide evidence for gene transfers and protein re-targeting as relatively early events in the evolution of plastid symbioses.

## Graphical abstract

**Graphical abstract:**
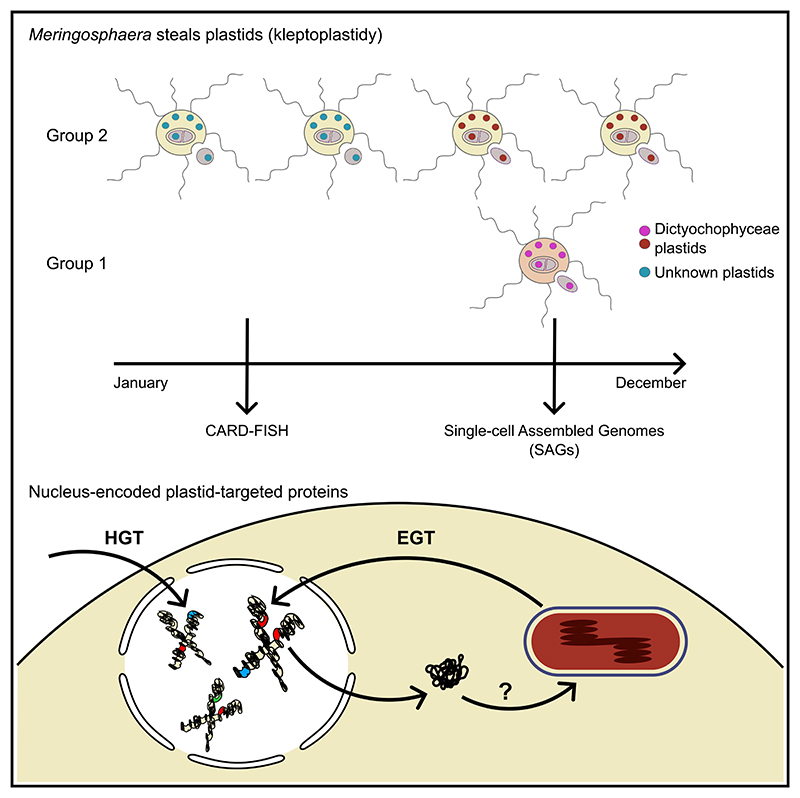

## Introduction

The evolution of plastids involved gains, losses, and replacements, spreading photosynthetic capabilities to most eukaryotic supergroups and creating a complicated pattern across the tree of life.^1^ Eukaryotes first acquired photosynthesis in an ancestor of Archaeplastida through endosymbiosis with cyanobacteria, establishing the primary plastids.^2–4^ Subsequent endosymbioses between eukaryotes have spread these plastids into new lineages on multiple independent occasions.^5,6^ In addition to the acquisition of these permanent organelles, plastids can be acquired temporarily either by symbiotic interactions with microalgal whole cells (photosymbiosis) or by retaining plastids from prey species by kleptoplastidy, which enables the host to transiently acquire photosynthetic capabilities. Kleptoplastidy is increasingly recognized as a common interaction in aquatic ecosystems, occurring in a broad range of host species, including a few multicellular eukaryotes such as the saccoglossan sea slugs,^7,8^ some marine flatworms,^9^ and a diverse list of protist hosts (including foraminiferans,^10^ ciliates,^11,12^ and dinoflagellates^13,14^). Kleptoplastidic interactions can be temporarily stable and thus offer unique insights into the establishment of plastids.^15^

Kleptoplastidy is a temporary association because the host cells cannot maintain stolen plastids indefinitely. In permanent plastids, a vast amount of protein import from host-encoded genes is required to compensate for their highly reduced plastid genomes.^16^ The imported proteins have mixed origins: some are the products of endosymbiotic gene transfer (EGT), others co-opted host proteins, and still others originate from horizontal gene transfers (HGTs).^17,18^ In the absence of this suite of imported proteins, kleptoplasts eventually degrade, and so the host must re-acquire plastids.^19^

Although some host species do little to counter the degradation of their kleptoplasts, other species actively support their stolen organelles and, in some instances, dramatically extend their survival. For example, the Antarctic Ross Sea dinoflagellate (RSD) can maintain its haptophyte-derived kleptoplasts for at least 30 months without replacement,^20^ probably by targeting host-encoded proteins to the kleptoplasts and thereby providing some of the necessary proteins.^21^ Interestingly, the nuclear genes that are targeted to the kleptoplasts often originated from HGT and not EGT, which aligns with the growing evidence of the importance of HGT for symbiotic integration.^6,22,23^ To date, the presence of kleptoplast-targeted proteins in the host nucleus has only been documented in three cases: in RSD, in another dinoflagellate, *Dinophysis acuminata*, that has cryptophyte-derived kleptoplasts,^24^ and in the euglenozoan *Rapaza viridis* that has green alga-derived kleptoplasts.^25^ However, the survival of kleptoplasts can be extended with a variety of other strategies. For instance, sacoglossan sea slugs exhibit shielding strategies that protect their kleptoplasts from potentially damaging light intensity.^26–29^ Similarly, some benthic foraminifera have been hypothesized to use behavioral strategies to protect their kleptoplasts from high light.^30^ Alternatively, in karyoklepty, the nucleus of the prey is also retained, and it remains transcriptionally active and services the kleptoplasts by providing the necessary nuclear-encoded proteins. This was first documented in the ciliate *Mesodinium rubrum* (also called *Myrionecta rubra*),^31^ where the cryptophyte nuclei are retained for up to 30 days, and has since also been identified in the dinoflagellate *Nusuttodinium aeruginosum.^32^* The diversity within host strategies used to control and extend kleptoplast survival remains an open question, as well as how many of these strategies involve host-encoded genes.

The import of host-encoded proteins into plastids is typically considered a benchmark of permanent and stable plastids. The prediction of protein import in kleptoplastidic systems indicates that a degree of genetic integration can already be achieved in temporarily retained plastids. It has been proposed that kleptoplastidic interactions controlled by host-encoded genes could represent a “tipping point” to stable genetically integrated plastids.^15,21^ Furthermore, the acquisition of foreign genes (e.g., HGT) by a kleptoplastidic host could provide pre-adaptations to future plastids. In this way, kleptoplastidy fits within the “shopping bag” model of plastid evolution,^33^ in which the serial uptake of symbionts provides necessary pre-adaptations required for permanent plastid establishment. Under this model, protein import establishes prior to permanent plastids and acts as an evolutionary ratchet that enables plastid fixation.^34–36^

In this study, we investigated a little-known marine protist, *Meringosphaera*, and the autofluorescent “green bodies” consistently observed internally. *Meringosphaera* is a globally distributed genus with distinctive undulating silica spines,^37,38^ which can reach dominance in planktonic assemblages.^39,40^ Obiol et al.^41^ reported that *Meringosphaera* (referred to as Centrohelida-sp1) was “the most widespread and abundant ASV” (amplicon sequence variant) during the Malaspina 2010 Circum-navigation Expedition. Despite this, *Meringosphaera* remains remarkably understudied. First characterized in 1902,^42^ it has historically been treated as a chrysophyte-related alga due to the consistent presence of green bodies presumed to be chloroplasts.^38,43^ Recently, the first *Meringosphaera* 18S rDNA sequence was obtained, which led to its re-classification as a centrohelid (Haptista: Centroplasthelida), in the environmental marine clade NC5.^44^ Importantly, there are no permanent plastids known in centrohelids,^45^ though there are two suggested cases of kleptoplastidic freshwater species^46^ and endosymbiotic *Chlorella* have been found in the centrohelid *Acanthocystis turfacea.^47^* Centrohelids are typically not considered to have been ancestrally photosynthetic.^48^ The origin of the green bodies in *Meringosphaera* is, therefore, unknown.

Since *Meringosphaera* is uncultivated, we used a suite of culture-independent approaches on surface water collected monthly from the North Sea (West coast of Sweden) to characterize the fluorescent bodies observed in the cells. Following manual isolation of *Meringosphaera* cells, we generated single-cell amplified genomes (SAGs) to study the genetic identity of the plastids and to search for evidence of genetic integration via host-encoded plastid-associated genes. Specific quantitative PCR (qPCR) assays were designed for environmental monitoring of *Meringosphaera* host and plastids over 14 months of sampling. Catalyzed reporter deposition-fluorescence *in situ* hybridization (CARD-FISH) was used with confocal microscopy to confirm the 18S rRNA of the host *Meringosphaera* and to visualize the presence/absence of plastids. Overall, the results suggest that *Meringosphaera* exhibits selective kleptoplastidy with evidence of host-encoded plastid-associated genes. The exact identity of the kleptoplasts depends on the season and the host lineage. Except for the euglenozoan *Rapaza viridis*, this is the only other known case of genetic integration in a kleptoplastidic host outside of dinoflagellates. This is particularly significant because centrohelids, unlike dinoflagellates but like *Rapaza viridis*, are not ancestrally plastid-bearing, and thus these host-encoded genes arose in a nuclear genome that had not previously co-evolved with a plastid.

## Results

### Origin and diversity of *Meringosphaera* plastid association

**Figure 1:**
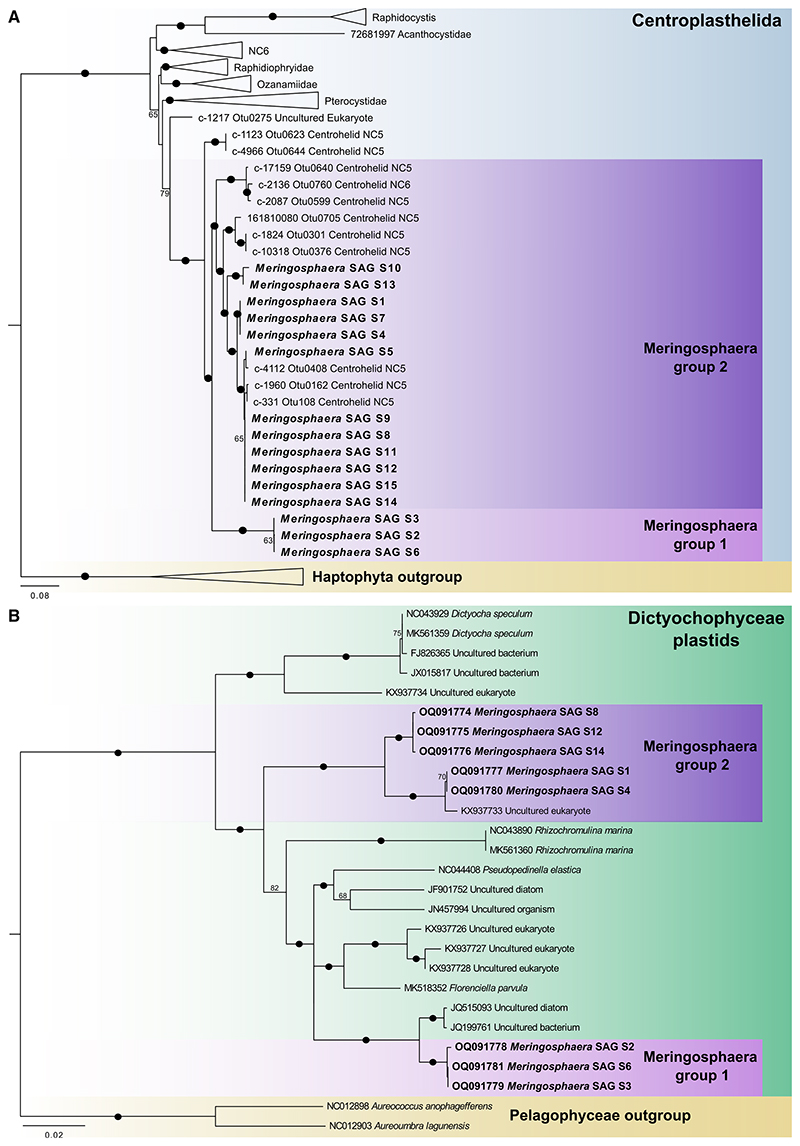
Diversity and phylogeny of the *Meringosphaera* host and its plastid from the Swedish west coast (A) Maximum likelihood tree of selected representative concatenated 18S and 28S rDNA sequences showing *Meringosphaera* within the Centroplasthelida. The tree was reconstructed with a GTR model, with 4 gamma categories, and support values correspond to rapid bootstrap with 1,000 replicates. The two groups are designated as group 1 and group 2, these two groups corresponded to clustering the *Meringosphaera* 18S rDNA sequences by >97% identity (the separate 18S and 28S phylogenies are available in the Figshare repository D2.1). The *Meringosphaera* sequences written in bold were generated by this project, and all other previous sequences are unidentified environmental sequences. Haptophyte sequences were used as an outgroup. (B) Maximum likelihood tree of selected cultured and representative 16S rDNA sequences showing the *Meringosphaera* plastids within the Dictyochophyceae plastids. See Figure S1 for the phylogenies of the additional plastid genes *psbA* and *rbcL*. The tree was reconstructed using a TIM3 + F + I + G4 model, and support values correspond to ultrafast bootstrap values from 1,000 replicates. The *Meringosphaera* plastid sequences written in bold were generated by this project. Pelagophyceae sequences were used as outgroup. In both trees, support values over 50% are shown on the trees, with values over 90% represented by a black circle on the branch. See STAR Methods for details regarding the tree formation.

We used 15 manually isolated *Meringosphaera* cells, collected in October and November 2018, to perform individual genome amplification and assemble SAGs. Using the previously reported *Meringosphaera* 18S rDNA sequence as query (GenBank: MZ240752^44^), we found by BLASTn a highly similar 18S rDNA sequence (95%–99% identity) in every SAG. Using two centrohelid 28S rDNA sequences as queries (GenBank: JQ245080 and AY752993), we further identified a 28S rDNA sequence in every SAG. When placed in a concatenated 18S and 28S rDNA phylogeny alongside representative centrohelid sequences (Figure 1A), the *Meringosphaera* sequences and a few environmental sequences formed a well-supported monophyletic clade (bootstrap percentage[BP] = 95%). The SAGs were split into two groups (“group 1” and “group 2,” BP = 100% and 92%, respectively), representing previously unknown genetic diversity.

To assess whether the green bodies commonly observed within *Meringosphaera* correspond to full endosymbiont cells, we searched for any additional 18S rDNA sequence in the SAGs using a diverse set of reference sequences (see STAR Methods). In only two SAGs did we identify a second 18S rDNA sequence (S5 and S7, both with 100% sequence similarity with the green algae *Nannochloris sp*.); however, given that it was rare, inconsistent across the samples, and mismatched to the plastid identity (see below), we considered it unlikely to correspond to the observed fluorescent bodies. In the remaining 13 SAGs, apart from the *Meringosphaera* 18S rDNA, no other 18S rDNA sequences were found. This suggests the absence of another eukaryotic nucleus (from, e.g., prey or photosymbionts), and thus that the fluorescent bodies likely correspond to plastids.

The identity of the plastids was determined by extracting 16S rDNA sequences from the SAGs. Despite that all picked cells appeared to contain green fluorescent bodies, we only found 16S rDNA plastid sequences in eight of the 15 SAGs. A 16S rDNA phylogeny showed that all eight plastid sequences are closely related to plastids from Dictyochophyceae and a few environmental sequences but were clearly distinct from the few described species (Figure 1B). The plastid phylogeny based on maximum likelihood showed a similar pattern to the host 18S rDNA phylogeny, with two distinct and well-supported clades (group 1 plastids BP = 97%, group 2 plastids = 100 %). In order to confirm the pattern given by the 16S rDNA plastid phylogeny, we also constructed phylogenies for two other plastid molecular markers: the large subunit of ribulose 1,5-bisphosphate carboxylase/oxygenase (*rbcL*) and the D1 protein of photosystem II (*psbA*). Both genes were only recovered in a subset of the SAGs (but fully overlapping with the eight SAGs with plastid 16S rDNA sequences): *rbcL* in 11 SAGs and *psbA* in nine SAGs (summarized in Table S1). Congruent with the 16S rDNA phylogeny, the *rbcL* and *psbA* sequences from the *Meringosphaera* plastids were most closely related to Dictyochophyceae and partitioned into two distinct groups (Figure S1), providing additional evidence for the plastid origin and that distinct plastids are found in the two major host groups. Interestingly, for all three of the plastid genes, the branching pattern is congruent with the 18S rDNA phylogenies, i.e., the group 1 hosts have one type of plastid, whereas the group 2 hosts have the other.

### Seasonal dynamics of *Meringosphaera* and its plastid

**Figure 2:**
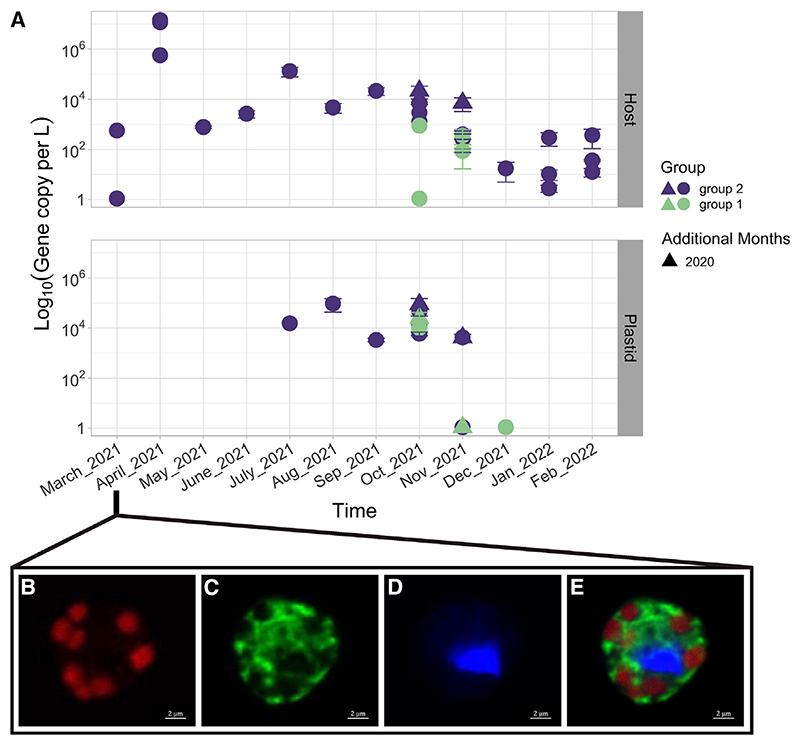
Seasonal dynamics of *Meringosphaera* and its plastids (A) Abundance of *Meringosphaera* group 1 and 2 and respective plastids detected in the monthly sampling from the Swedish west coast. The top panel shows the number of 18S rDNA gene copies L^−1^ for the *Meringosphaera* group 1 and group 2 and the lower panel shows the respective number of *rbcL* gene copies L^−1^ forthe plastid group 1 and group 2. The group identity of the hosts and plastids is indicated bythe colorofthe points.The abundance is reported as the Log of the gene copies L^−1^. The samples were taken monthly between March 2021 and February 2022, in addition two extra samples were taken in October and November 2020, and these are indicated by triangle-shaped points. There is variation in the number of locations sampled, which is why the number of points varies between the months (see Figure S2 and STAR Methods for details). If the target was detected in some but not all of the technical replicates, the sample was marked as detected but not quantifiable and was plotted with a value of 1.1. Data are represented as the mean ± SEM. See also Figure S3 for the hydrographic conditions at the sampling stations. (B–E) Images of *Meringosphaera* and its kleptoplasts collected in April 2021 from the Å17 sampling station and visualized by CARD-FISH, showing that the plastids are numerous and intact despite the lack of detection in the qPCR assay. The cells were imaged on a confocal microscope with the 405, 488, and 633 nm lasers. In all panels, the green signal is from the Alexa 488 dye bound to the Mer482 probe that was designed to specifically target *Meringosphaera* 18S rRNA, the blue signal is from the nucleic acid stain DAPI, and the red signal is from chlorophyll *a* autofluorescence. (B)–(E) are images taken from the same cell, (B)–(D) show each channel individually, and (E) shows the overlay of the three channels. Scale bars, 2 μm. Video S1 is composed of the z stack images taken from this same cell.

To investigate the seasonal dynamics of *Meringosphaera* and its plastids, we surveyed over a year of samples and three locations with targeted qPCR assays that distinguish between group 1 and group 2 for both the host *Meringosphaera* 18S rDNA and the plastid-encoded *rbcL* genes. The assay was applied to whole water samples, meaning that the plastid *rbcL* detection could originate from plastids either inside *Meringosphaera* or inside free-living Dictyochophyceae. The qPCR assay was designed based on the SAG data from October to November 2018, and as such the oligonucleotides could be biased toward the groups found during that time of year. The 18S rDNA results (Figure 2A) show that *Meringosphaera* group 2 was present throughout the year (mean ~ 1.1 × 10^6^ gene copies L^−1^), but gene abundance varied throughout the months (ANOVA, F_11,45_ = 8.53, p < 0.001). The highest gene copies (~1.5 × 10^7^ gene copies L^−1^) were detected in April, in what could be a spring bloom. Otherwise, in general, the gene abundances were higher (~1.3 × 10^5^ gene copies L^−1^) in late summer after which they decreased through autumn down to very low frequencies in winter (range of 3–374 gene copies L^−1^). By contrast, the *Meringosphaera* group 1 host was only detected in October and November of 2021. The presence of group 1 host in 2021 and not 2020 could have biological reasons but may also be explained by sampling strategy given that in 2020 only one of the three sites was sampled.

The seasonal dynamics of the plastids only partially corresponded to that of the *Meringosphaera* host (Figure 2A). Neither plastid groups were detected from January to June, and in July, August, and September only the group 2 plastid was detected. The identified plastids from the SAGs were, therefore, only detected in the second half of the year. In the first half of the year, the group 2 *Meringosphaera* was either aposymbiotic or switched to a different plastid that was not detected by our assays. Either scenario suggests that this is kleptoplastidy and not a permanent plastid. By contrast, the group 1 plastid displayed a similar temporal dynamic with the group 1 host as both were only detected between October and December. However, group 1 hosts and plastids showed spatial separation as they were detected in different sampling locations that differ in their hydrological conditions (Figures S2 and S3). The data suggest that the geographical distribution of the group 1 host and plastid is complicated, with potential migrations into our sampling locations for only 2–3 months of the year. Unfortunately, we do not have sufficient sampling data to form a complete picture of the changing distribution of group 1—future work will need to address this. Interestingly, qPCR data for 2 consecutive years in October and November (2020 and 2021) showed consistency in gene copy number and plastid detection (Figure 2A). The plastid identity at this time of year was also consistent with SAGs from earlier samples in October and November 2018. This level of consistency suggests that the fluctuations in plastid identity could be repeating annually, and therefore seasonal dynamics might play an important role in the *Meringosphaera* kleptoplastidy (although a longer time series is needed to confirm these preliminary observations). No pattern between basic environmental conditions and *Meringosphaera* host or plastid gene abundances was found (Figure S3).

### Visualization of the *Meringosphaera* plastids

To test whether the group 2 *Meringosphaera* was aposymbiotic from January to June or had instead switched plastid, we used CARD-FISH and confocal microscopy to visualize individual *Meringosphaera* cells. We designed a specific CARD-FISH probe for identifying the *Meringosphaera* host, which in combination with chlorophyll autofluorescence enabled the visualization of potential photosynthetic plastids. We used samples from the spring bloom of April 2021, a time when our qPCR assay detected high copies of the group 2 *Meringosphaera* host (e.g., 10^5^–10^7^ gene copies L^−1^) but both plastid groups were below detection.

The CARD-FISH probe hybridized only to appropriately sized cells with the expected morphology and cell diameters (4–9 μm diameter spherical cells) indicating a successful CARD-FISH procedure. We did not detect the characteristic undulating spines of *Meringosphaera*, but this was expected given the acidic steps in the CARD-FISH procedure (see STAR Methods). The CARD-FISH images (Figures 2B–2E) clearly show the presence of multiple and intact plastids within a positively hybridized *Meringosphaera* cell. The chlorophyll *a* autofluorescence and 18S rRNA probe occupied distinct regions within the *Meringosphaera* cell, revealing good intracellular specificity (Figures 2B and 2C). The plastids appear located to the edge of the cell, in a very similar arrangement to previous observations of *Meringosphaera* cells with plastids (e.g., Figures 1A and 1B in Zlatogursky et al.^44^). The z stack images (Video S1) confirmed that the plastids were positioned within the cell and not located either above or below. DAPI only stained the *Meringosphaera* nucleus (Figure 2D), but no potential nuclei associated with the plastids, supporting the SAG analysis that did not find a second nucleus. The clear presence of plastids within some *Meringosphaera* cells in April 2021 but a lack of detection by our specific qPCR assay suggests that *Meringosphaera* might be able to switch plastids.

### Variation in plastid integrity and gene repertoire

**Figure 3:**
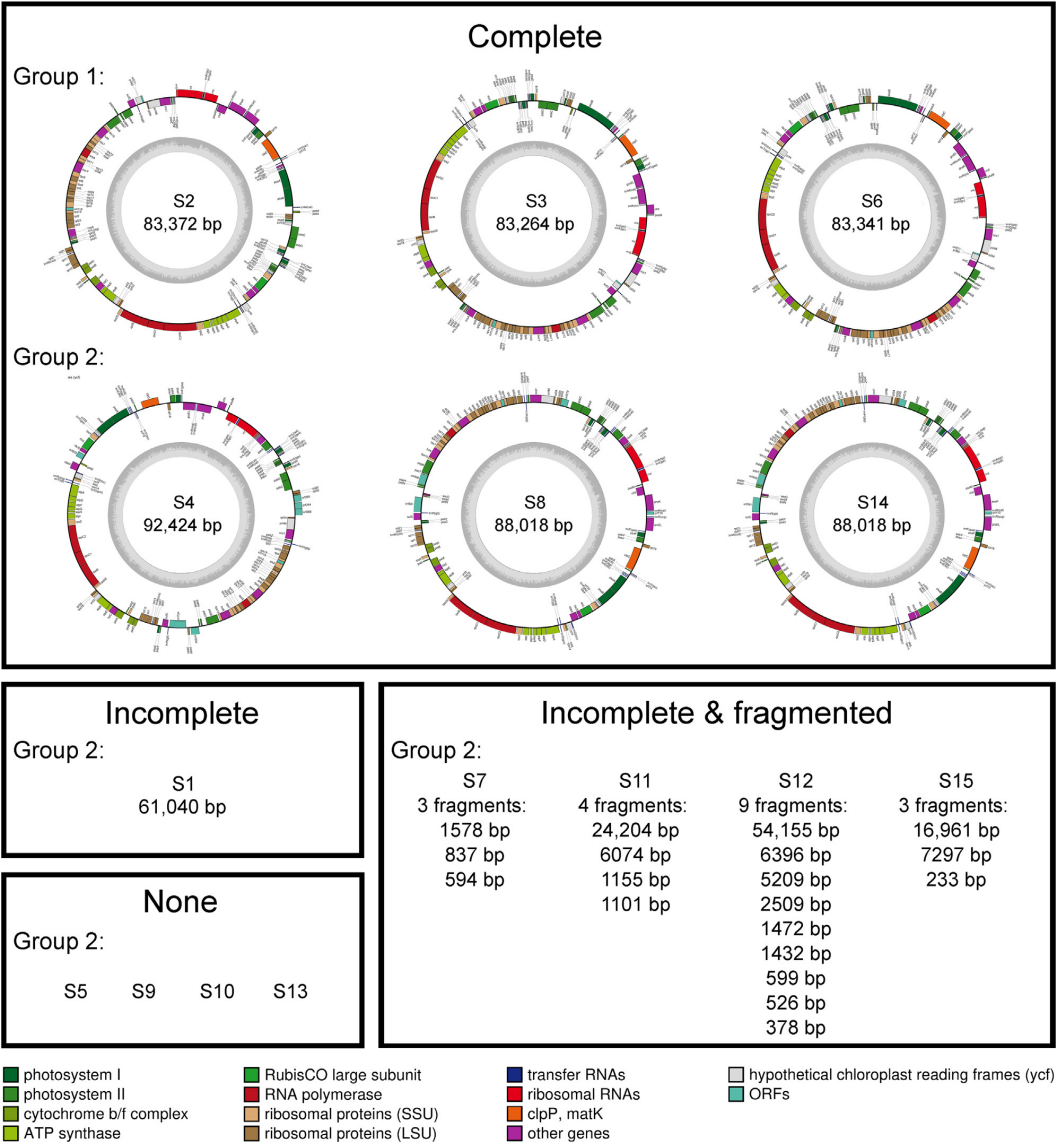
Variation in the completeness of the plastid genomes from group 1 and group 2 *Meringosphaera* SAGs The plastid genome maps are shown for genomes predicted to be complete and circular. For the incomplete genomes, the number of fragments and size of each fragment is written per SAG. The function of the predicted ORFs in the complete genomes are indicated by their colour as described in the legend.

Plastid contigs were recovered from a total of 11 SAGs, with six mapping into circular genomes. The completeness of the plastid genomes varied for the other SAGs: five possessed incomplete plastid genomes, four of which were fragmented across multiple contigs, and four SAGs lacked plastid sequences (Figure 3; Table S1). The six complete plastid genomes, recovered from both *Meringosphaera* group 1 and 2, were annotated and compared with the closest available Dictyochophyceae with reference plastid genomes (all free-living). The SAG plastid genome sizes varied according to group identity (Figure 3; Table S1). The group 1 genomes were slightly smaller with a mean size of 83,326 bp, whereas the group 2 genomes had a mean of 89,487 bp, but the number of predicted open reading frames (ORFs) were similar between the two groups varying between 137 and 141. Both the genome size and predicted gene number are lower than those of the reference Dictyochophyceae plastid genomes, which have plastid genomes ranging from 108,152 to 140,025 bp with 144–159 predicted genes.^49^

**Figure 4:**
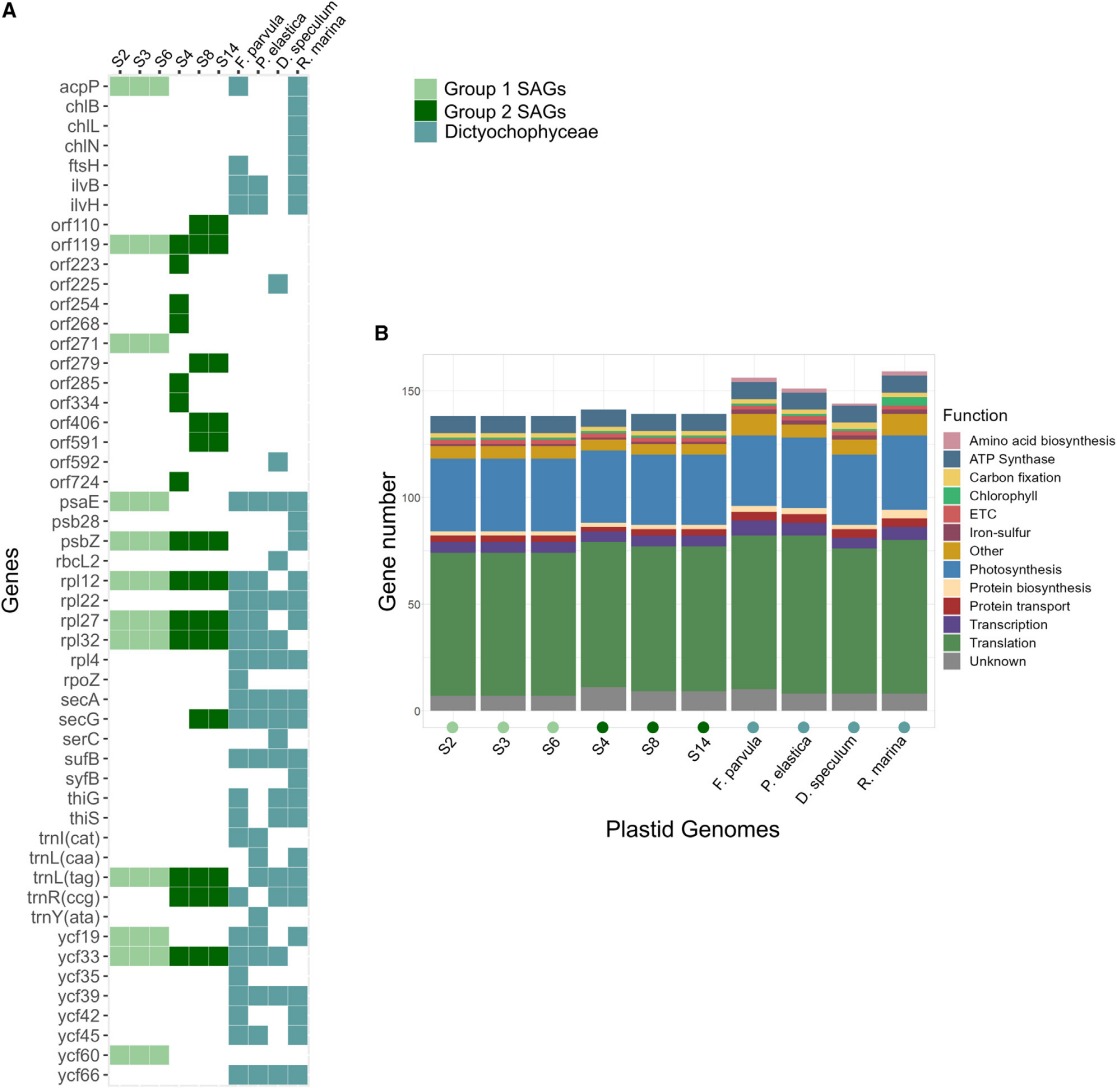
Comparison of the gene repertoires of the *Meringosphaera* plastid genomes with free-living Dictyochophyceae (A) Presence/absence of annotated genes between the six complete SAG plastids and four free-living photosynthetic Dictyochophyceae. Only genes that are not present in all sampled genomes are shown. The full set of plastid genes is available in Figure S4. The background color of the squares highlights the identity of the plastids (pale green, group 1 SAGs; dark green, group 2 SAGs; and pale blue, representative Dictyochophyceae). (B) Bar plot showing the predicted function of the annotated genes, with color denoting the functional group as described in the legend. The same genomes are compared as in (A), and the color circle below the genome name highlights the identity using the same color scheme as for (A).

The functional repertoire of the plastids found in *Meringosphaera* shows that they retain the capacity to perform photosynthesis (Figures 4 and S4). In particular, they retain the core components of both photosystem I and II (*psa*- and *psb*- genes), carbon fixation (*rbcL* and *rbcS*), cytochrome b6/f complex (*pet*- genes), ATP synthase (*atp*- genes), and chlorophyll biosynthesis (*chlI*). Compared with the Dictyochophyceae plastids, six genes are lacking in all of the *Meringosphaera* plastids (Figure 4A). These missing genes span a range of functions: ribosomal proteins (*rpl22* and *rpl4*), protein translocase (*secA*), iron-sulfur cluster assembly (*sufB*), and two uncharacterized genes (*ycf39* and *ycf66*). All of these genes have been lost from the plastid genome of other algae, for example, *rpl22* has been lost within the Alveolates,^50^ *rpl4* and *secA* lost in Pelagophyceae,^51^ *sufB* lost in *Pteridomonas^52^* and transferred to nucleus in green algae,^53^ *ycf39* lost in Eustigmatophytes and *Ochromonas*,^54^ and *ycf66* lost in some diatoms.^55^ In addition, none of the plastids found in *Meringosphaera* have any of the three amino acid biosynthesis genes *ilvB, ilvH*, and *serC*, whereas the known Dictyochophyceae plastids have at least one copy. Furthermore, one gene, *orf119*, is present in all of the *Meringosphaera* plastids but not in Dictyochophyceae and encodes a 50s ribosomal L22 like-protein. It is potentially a derivative of the *rpl22* gene that is missing in all the *Meringosphaera* plastids. The ten genes with a shared pattern across the *Meringosphaera* plastids compared with the free-living Dictyochophyceae (i.e., *rpl22*, *rpl4*, *secA*, *sufB*, *ycf39*, *ycf66*, *ilvB*, *ilvG*, *serC*, and *orf119*), indicate a level of convergence in the putative *Meringosphaera* kleptoplasts since plastid groups 1 and 2 are separated by free-living species.

The gene content within the two main plastid groups of *Meringosphaera* also showed patterns of gene specificity (Figures 4A and S4). Group 1 has five genes not found in group 2: *acpP*, which encodes a cofactor of fatty acid synthesis; the accessory subunit of PSI, *psaE;* the plastid-encoded member of the TIC20 family, *ycf60;* and two genes of unknown function (*orf271* and *ycf19*). The presence of the plastid copy of the TIC20 transporter, *ycf60*, within group 1 is particularly interesting because this could be part of a protein import machinery into the plastid. TIC20 has been found to be capable of forming an independent channel in the chloroplast inner membrane that does not require other TIC proteins to function.^56^ By contrast, group 2 had one specific gene, *trnR* a tRNA-arginine, that is involved in translation.

### Host-encoded plastid-associated genes

To determine whether kleptoplastidy in *Meringosphaera* shows evidence of host genetic integration, we searched for host-encoded genes of plastid-associated pathways. The 15 individual SAGs were co-assembled based on a 99% 18S rDNA identity threshold to improve genome coverage and to help address the biases of the multiple displacement amplification (MDA) process (Table S1; the STAR Methods detail which SAGs were coassembled together). Both SAGs with and without plastids were co-assembled. This led to the assembly of three COSAGs (COSAG1, COSAG2a, and COSAG2b): COSAG1 belongs to group 1 of the 18S rDNA phylogeny, and COSAG2a and COSAG2b belong to group 2. As expected, the co-assemblies had higher completeness than the individual SAGs (Table S2). We searched for 31 well-characterized nuclear-encoded proteins that function in key plastid pathways even in non-photosynthetic species (e.g., isoprenoid synthesis, iron-sulfur cluster biosynthesis, and protein import).^21,57^ In addition, we searched for 35 metabolic transporters that have been previously identified as kleptoplast-targeted.^25^ For each protein candidate we (1) identified its genomic context to ensure that it was within a *Meringosphaera* contig and not a contaminant, (2) identified the phylogenetic origin of the candidate protein, and (3) predicted the subcellular localization by searching for a plastid-targeted signal (see STAR Methods for further details). For the metabolic transporters, only candidates with predicted plastid targeting were kept to avoid the inclusion of homologous copies functioning in other cellular compartments.

**Figure 5:**
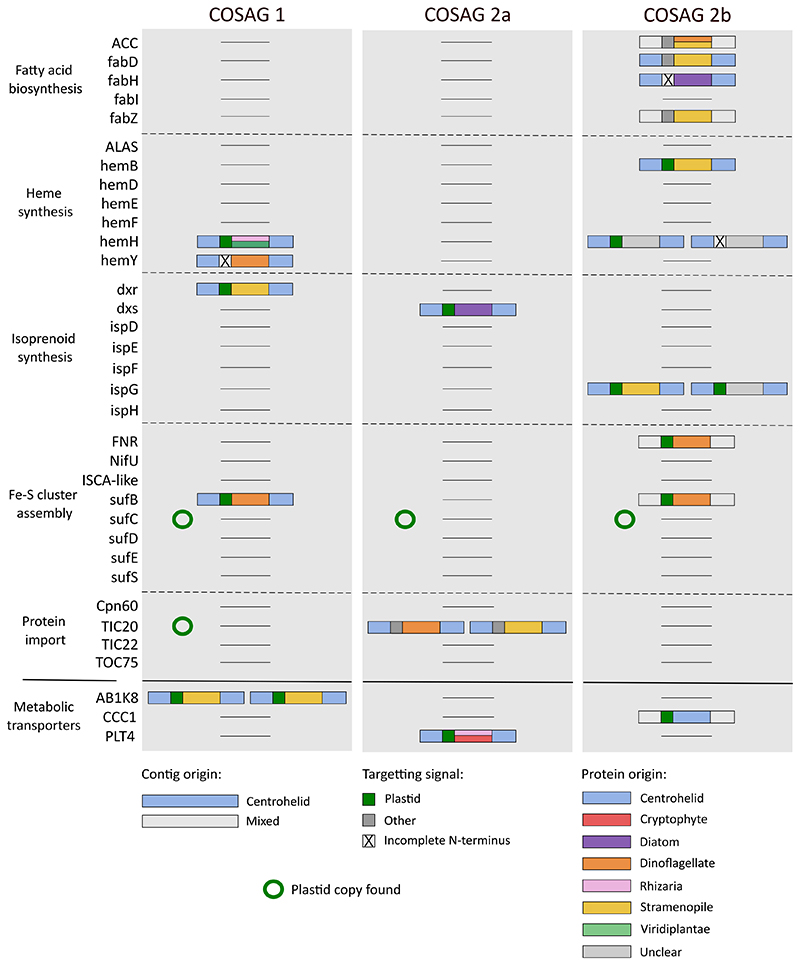
Predicted candidates for host-encoded plastid-associated proteins in the co-assemblies Each column shows the data for one of the co-assemblies, and each row represents one of the host-encoded plastid-associated proteins, which have been grouped by their function. First, there are the 31 known host-encoded plastid-associated proteins that we searched for, and at the bottom, the three metabolic transporters identified with predicted plastid targeting. The color of the larger box indicates how confident we are in the contig origin; blue shows a confident centrohelid identity, and gray represents a mixed identity that includes centrohelids among others. The small box indicates the predicted targeting signal: dark green indicates predicted plastid targeting, gray indicates predicted targeting to other cellular compartments, and a cross is shown when the N terminus was incomplete and targeting could not be predicted (see Table S3 and Figure S5). The central box shows the phylogeneticorigin of the candidate protein, with colors representing different groups. For the metabolic transporters, only those with predicted plastid targeting were kept. If a plastid copy of the gene was found, a green circle is shown.

In total, we found 22 candidates corresponding to *Meringosphaera* host-encoded plastid-associated proteins (Figure 5; summarized in Table S3). These proteins were found across the three COSAGs and across a diverse range of functions. 14 of the 22 candidates were predicted to have a plastid-targeting signal with a clear N-terminal extension (Figure S5; Table S3). Four homologs to metabolic transporters were predicted, corresponding to three different transporters. Of particular interest is PLT4, a probable sugar transporter predicted in COSAG2a, which could function to translocate fixed carbon into the cytosol. Dinoflagellates and stramenopiles were the main phylogenetic origins of the plastid-targeted proteins (six proteins were predicted as dinoflagellate in origin and nine proteins as stramenopile). Since Dictyochophyceae are stramenopiles, these nine proteins could have originated from EGT, although we could not determine a more precise origin to a specific stramenopile group. It is unclear why six proteins have a dinoflagellate origin, but their genes could have arisen from HGT from other food sources or via a hypothetical second kleptoplast during plastid switching.

Some of these plastid-associated proteins are known to be encoded in either the plastid or nuclear genome in various species, so these need to be considered alongside the plastid genome content. For example, the iron-sulfur cluster assembly genes *sufB* and *sufC* are typically encoded on the plastid genome, whereas the remaining genes of the pathway are nuclear-encoded.^58^ The plastid genomes of the SAGs contained *sufC* but not *sufB* (Figures 4 and S4), but both COSAG1 and COSAG2b had a nuclear candidate *sufB* gene. Similarly, although the group 1 plastid genomes encoded the plastid TIC20-homolog (*ycf60*), the nuclear TIC20 candidate was only found in group 2 (COSAG2a). It remains to be confirmed if these patterns of complementarity hold with more complete data, but both of these examples suggest that host-encoded proteins can substitute missing plastid-encoded functions.

## Discussion

*Meringosphaera* is a poorly studied marine genus that can reach high abundance and has been known for a century to harbor photosynthetic green bodies. Here we identified these commonly observed bodies and investigated the nature of their partnership. Our analyses based on single-cell genomic data and microscopy found no evidence of endosymbiont nuclei, which suggests that the green bodies are isolated plastids and not whole endosymbiotic cells. The analysis of the SAG data revealed two main groups of *Meringosphaera* harboring different Dictyochophyceae plastids. Our monthly environmental monitoring spanning over a year uncovered seasonal dynamics of both host and plastid. In particular, the group 2 plastid was not detected between January and June, which we hypothesize is due to plastid switching based on the observation by fluorescence of plastids in *Meringosphaera* cells collected during this time (Figure 2). The lack of plastid detection combined with the lack of plastid markers in four of the SAGs, suggest that the plastids in *Meringosphaera* are kleptoplasts stolen from Dictyochophyceae prey. Dictyochophyceae are photosynthetic stramenopiles with red algal-derived plastids.^49^ There are only two previous reports of symbiotic Dictyochophyceae: (1) as kleptoplasts found in the dinoflagellate host *Dinophysis mitra* (also known as *Phalacroma mitra*),^59^ and (2) in the dinoflagellate *Podolampas bipes^60^* where endosymbiotic cells are apparently vertically transmitted to daughter cells. Unfortunately, there are no plastid genomes from either of these examples, so we cannot compare *Meringosphaera* kleptoplast genomes to other dictyochophyte symbionts.

The presence of kleptoplasts in *Meringosphaera* is consistent with historical observations that have reported cells both with photosynthetic bodies^38,42,43^ but also more rarely without.^61,62^ Furthermore, these few available microscopic observations reported contrasting colors of the *Meringosphaera* photosynthetic bodies, including green^42,63^ and golden,^43^ which is consistent with the presence of a cryptic diversity of hosts that harbor different kinds of kleptoplasts or with plastid switching. Seasonal dynamics within kleptoplastidy similar to the type we report here have been documented before. For example, temporal changes were found in the identity of the green algal kleptoplasts within the sea slug *Plakobranchus ocellatus.^64^* The ciliate *Mesodinium* spp. was also found to switch between red and green-pigmented cryptophyte plastids throughout the year.^65^ However, it has not yet been demonstrated whether plastid switching in kleptoplastidy can actively select the “optimum” plastid for a set of given conditions or whether it is always passive and responds to prey availability. The latter would be akin to the discovery that symbiont biogeography dictates the association in some marine photosymbioses.^66^

The plastid genomes of *Meringosphaera* varied in both their recovered integrity and gene content. The variation in integrity could be an artifact of the MDA process, which is known to lead to uneven amplification and coverage that can prevent proper assembly.^67^ Alternatively, the variation might reflect different stages of kleptoplastidy as the plastids eventually degrade without the required nuclear support.^9^ This could explain the four SAGs with no plastid sequences, which might have been the oldest cells containing highly degraded plastids with insufficient intact DNA for the MDA process to recover. Among the differences in gene content, the most striking was the presence of the plastid homolog of *TIC20* within the group 1 plastids. Intriguingly, a host-encoded *TIC20* gene was identified in COSAG2a. Combined, this indicates that two of the three co-assembly groups of *Meringosphaera* have a possible protein import channel that has been demonstrated to permit some protein import without other subunits.^56^

The predicted host-encoded plastid-associated proteins in *Meringosphaera* are one of the few examples of putative genetic integration shown in kleptoplastidy.^21,24,25^ The candidates for the host-encoded plastid associated-proteins were part of different pathways, but no pathway was completely recovered in our analysis of plastid pathways. This could be indicative of mosaic pathways, where enzymatic steps occur in different cellular compartments. This has, for example, been found previously in the heme synthesis pathway of some dinoflagellates and apicomplexans, where the first step utilized the mitochondrial version of the 5-aminolevulinic acid synthase (ALAS) enzyme but the later steps with the hemB-E enzymes occurred separately in the plastid.^68^ It is possible that some of the pathways within *Meringosphaera* are similarly mosaic and that some stages occur in other cellular compartments. Alternatively, these pathways might be incomplete, which would contribute to the eventual degradation of the kleptoplasts, or be due to the partial nature of the SAGs.

We do not yet know whether the host-encoded plastid-associated proteins in *Meringosphaera* are successfully targeted to the kleptoplasts where they function. Nonetheless, finding host-encoded genes putatively associated with kleptoplasts is significant for two main reasons. First, it shows that low levels of EGT and possibly HGT may take place in a fluctuating kleptoplastidy where the kleptoplasts are sourced from different preys. By contrast, *Rapaza viridis* and the dinoflagellate hosts with kleptoplast-targeted proteins have remarkably stable kleptoplastidy (over 30 months without renewal in RSD), and only one specialized prey species is known.^21,24,25^ Secondly, unlike in dinoflagellates but as in *R. viridis*, centrohelids are not ancestrally plastidbearing, and therefore, kleptoplast-associated gene transfers occurred in a naive genome with no pre-adaptations to plastid hosting. These observations suggest that low-level protein import can occur early-on as a mechanism to regulate plastid retention in kleptoplastidy. The finding of protein import into the kleptoplasts of *Meringosphaera* is congruent with the predictions of the shopping bag model of plastid origin, where serial uptake of foreign genes from food facilitated the eventual fixation of a plastid.^33^ Furthermore, the predicted kleptoplast-targeted metabolic transporters are consistent with the targeting-ratchet model,^35^ which hypothesizes that transporters set up an evolutionary ratchet for plastid fixation. Overall, this work supports the hypothesis that protein import is a relatively early event that helps to stabilize plastids,^34–36^ and as such is a mechanism, rather than a consequence, of plastid establishment.

In conclusion, *Meringosphaera* offers an exciting opportunity to examine kleptoplastidy within centrohelids and so provides new insights, from a relatively under-examined eukaryotic group, into this important process for the evolution of photosymbiosis. Future work needs to identify the second putative kleptoplast in *Meringosphaera*, after which it could be used as a model to investigate plastid switching. Moreover, as a globally distributed and at times highly abundant species, the study of *Meringosphaera* is also potentially important for the marine ecosystems in which it plays a role. This study into the little-known centrohelid *Meringosphaera* demonstrates the insights gained into important evolutionary transitions by continuing to explore the broad range of eukaryotic diversity.

## Star★Methods

Detailed methods are provided in the online version of this paper and include the following:

- KEY RESOURCES TABLE
- RESOURCE AVAILABILITY
  ○ Lead contact
  ○ Materials availability
  ○ Data and code availability
- EXPERIMENTAL MODEL AND SUBJECT DETAILS
  ○ Sampling
  ○ Culturing attempts
  ○ Environmental Dictyochophyceae
- METHOD DETAILS
  ○ Single cell isolations & MDA
  ○ Sequencing
  ○ SAG assembly
  ○ Plastid genome assembly & comparisons
  ○ Co-assemblies & host-encoded plastid associated genes
  ○ CARD-FISH
  ○ Environmental monitoring with qPCR
- QUANTIFICATION AND STATISTICAL ANALYSIS

## Star★Methods

**Key Resources Table:** 

REAGENT or RESOURCE	SOURCE	IDENTIFIER
Chemicals, peptides, and recombinant proteins		
Paraformaldehyde	Serva	Catalog number: 31628
SeaKem LE agarose	Cambrex	Catalog number: *50004*
Lysozyme	Sigma Aldrich	Catalog number:12650-88-3
ProLong Diamond Antifade mountant	Thermo Fisher Scientific (Invitrogen)	Catalog number: P36961
Proteinase K	Qiagen	Catalog number: 19133
TaqMan buffer	Thermo Fisher Scientific (Applied Biosystems)	Catalog number: 4370074
Formamide	VWR	Catalog number: 1.09684.1000
Critical commercial assays		
REPLI-g UltraFast Mini kit	Qiagen	Catalog number: 150033
ExoProStar 1-Step Kit	GE Healthcare	Catalog number: US77702
DNeasy Plant kit	Qiagen	Catalog number: 69104
Deposited data		
*Meringosphaera* 18S rDNA sequences	GenBank	Accession numbers: OQ075975 to OQ075989
*Meringosphaera* 28S rDNA sequences	GenBank	Accession numbers: OR195151 to OR195157 & OR196762 to OR196769
*Meringosphaera* plastid 16S rDNA sequences	GenBank	Accession numbers: OQ091774 to OQ091781
*Meringosphaera* plastid *psbA* sequences	GenBank	Accession numbers: OQ078560 to OQ078568
*Meringosphaera* plastid *rbcL* sequences	GenBank	Accession numbers: OQ078569 to OQ078579
*Meringosphaera* complete plastid genomes	GenBank	Accession numbers: OQ161668 to OQ161673
Raw reads	NCBI Sequence Read Archive	under BioProject PRJNA917255, accession numbers SAMN32532880 to SAMN32532894
Data files - all the plastid contigs (both complete and incomplete), the host-encoded plastid-associated protein candidates, single gene trees, the assembled reads of the SAGs and COSAGS, and the qPCR results.	Figshare	https://doi.org/10.6084/m9.figshare.c.6313464
Custom scripts	GitHub	https://github.com/ MeganSorensen/Meringosphaera_SAGs
Oligonucleotides		
5’-CATATGCTTGTCTCAAAGATTAAGCCA-3’	Cavalier-Smith and von der Heyden^69^	Primer name: Thx25F
5’-CACACTTACWAGGAYTTCCTCGTTSAAGACG-3’	Cavalier-Smith and von der Heyden^69^	Primer name: Helio1979R
Software and algorithms		
TrimGalore v0.6.1	NA	www.bioinformatics.babraham.ac.uk/projects/trim_galore
Bbnorm (bbmap v38.08)	Bushnell^70^	https://github.com/BioInfoTools/BBMap
SPAdes v3.13.1	Bankevich et al.^71^	https://cab.spbu.ru/files/release3.13.1/manual.html
blast 2.10.1 +	Sayers et al.^72^	https://blast.ncbi.nlm.nih.gov/Blast.cgi
MAFFT v7.407	Katoh and Standley^73^	https://mafft.cbrc.jp/alignment/software/
trimAl v1.4.1	Capella-Gutierrez et al.^74^	https://github.com/inab/trimal
IQ-TREE v1.6.5	Minh et al.^75^	http://www.iqtree.org/
ModelFinder	Kalyaanamoorthy et al.^76^	http://www.iqtree.org/ModelFinder/
RAxMLv. 8.2.12	Stamatakis^77^	https://cme.h-its.org/exelixis/web/software/raxml/
GetOrganelle v1.7.3.3	Jin et al.^78^	https://github.com/Kinggerm/GetOrganelle
MFannot	NA	https://megasun.bch.umontreal.ca/cgi-bin/mfannot/mfannotInterface.pl
OGDraw	Greiner et al.^79^	https://chlorobox.mpimp-golm.mpg.de/OGDraw.html
R v.4.2.0	R Core Team^80^	https://cran.r-project.org/
RStudio	RStudio Team^81^	https://posit.co/download/rstudio-desktop/
BUSCO v5.3.1	Simao et al.^82^	https://busco.ezlab.org/busco_userguide.html
Prodigal v2.6.3	Hyatt et al.^83^	https://github.com/hyattpd/Prodigal
SequenceServer 2.0.0	Priyam et al.^84^	https://sequenceserver.com/
BWAv0.7.8	Li and Durbin^85^	https://bio-bwa.sourceforge.net/
IGVv2.4.2	Thorvaldsdottir et al.^86^	https://software.broadinstitute.org/software/igv/home
DEEPLOC 2.0	Thumuluri et al.^87^	https://services.healthtech.dtu.dk/services/DeepLoc-2.0/
TargetP 2.0	Almagro Armenteros et al.^88^	https://services.healthtech.dtu.dk/services/TargetP-2.0/
Other		
Alexa fluor 488	Thermo Fisher Scientific	Catalog number: A20000
47 mm diameter nominal pore size (5–15 μm) paper membrane filter	VWR	Catalog number: 516-0813
47mm-diameter 5μm polycarbonate hydrophilic membrane filters	Sigma Aldrich. (Millipore)	Catalog number: TMTP04700

### Resource Availability

#### Lead contact

Further information and requests for resources and reagents should be directed to and will be fulfilled by the lead contact, Megan Sørensen (Megan.Sorensen@hhu.de).

#### Materials availability

This study did not generate new unique reagents.

### Experimental Model And Subject Details

*Meringosphaera* cells were isolated from environmental samples by searching for characteristic *Meringosphaera* morphology: up to 13 undulating spines typically 16-25 μm long from a spherical body of 4-9 μm in cell diameter containing up to six green/yellow bodies and the cells are non-motile. The sampling strategy is detailed below. Once isolated, the cells were processed immediately and were not maintained in the lab.

#### Sampling

Samples were collected from surface waters at three stations off the West coast of Sweden: Anholt East (56°40’00” N, 12°07’00” E), Å17 (58°16’30”N, 10°30’48”E) and Släggo (58°15’30” N, 11°26’00” E) routinely sampled by the Swedish Meteorological and Hydrological Institute (SMHI) on the Research Vessel (R/V) Svea. On one occasion, October 2020, the Å17 sampling was replaced by sampling at a nearby station, Å16 (58°16’00” N, 10°43’30” E); which is 12 km away from Å17. Whole water samples were collected from the surface by bucket. The salinity is around 20 practical salinity units (PSU) at Anholt E, 31 PSU at Å17 and 24 PSU at Släggo. Surface water temperature at these locations typically ranges from ~0°C to 20°C over the year. Basic environmental data (temperature, salinity, dissolved nutrient concentrations) was taken from the SMHI website (https://sharkweb.smhi.se/hamta-data) corresponding to the dates and locations of our samplings (Figure S3). The samples were first stored on board the ship in the dark and then transported to the laboratory in an insulated, opaque container with icepacks; the total time between collection and arrival at the laboratory varied between 3 – 12 days.

#### Culturing attempts

We made several attempts to culture *Meringosphaera* but none of these were successful. These attempts included the inoculation of 1-10 *Meringosphaera* cells in 40 mm Petri dishes with 33 ppt artificial seawater or filter-sterilised water from the original habitat with or without additional nutrient media (soil extract, 0.025% cerophyl extract) at 4°C with or without light. Some dishes were separately cultured with Neobodo flagellates or the mixed protist community from original sample was added as potential food. The growth of *Meringosphaera* was never observed in any of these conditions and alive cells were not found in the original samples after two weeks.

#### Environmental Dictyochophyceae

The environmental diversity of Dictyochophyceae is an important factor for the *Meringosphaera* kleptoplastidy. We do not unfortunately have our own data of this, but we have performed searches for environmental Dictyochophyceae using the resources available:

First, regarding the Dictyochophyceae diversity in the environment at sampling sites, we have looked at the phytoplankton data taken by the SMHI monitoring program from the same stations we used (available at https://sharkweb.smhi.se/hamta-data). From this data, 4 Dictyochophyceae species have been identified by microscopy (*Apedinella radians*, *Dictyocha fibula*, *Dictyocha speculum* and *Pseudopedinella pyriformis*) and there were 3 additional genus-level identifications (*Dictyochales*, *Pseudochattonella and Pseudopedinella*). So there appears to be a range of Dictyochophyceae present, with 2 of the 4 known orders being identified. Unfortunately, the available data is abundance only and not sequence data.

Secondly, we have checked the *Tara Oceans* databases^89,90^ using the 16S rDNA plastid sequences as queries. Here there were some hits with 90-98.5% similarity. The upper part of this range is higher than the similarity to the known Dictyochophyceae species. Given that none of *Tara* stations are close to our sampling locations, a lack of exact matches could be due to environmental variation, and the top matches could represent either the free-living prey or additional *Meringosphaera* plastids. In addition, there are three environmental sequences that cluster with the *Meringosphaera* plastids in the 16S rDNA phylogeny, but again we do not know if these represent the free-living prey or not.

### Method Details

#### Single cell isolations & MDA

The samples for single cell isolation were collected in October and November 2018 from the West coast of Sweden (stations Å17 and Anholt E; Table S1 lists which SAGs were sampled from which stations). In the laboratory they were gravity filtered onto a 47 mm diameter nominal pore size (5–15 μm) paper membrane filter (VWR, Radnor, Pennsylvania, USA; Cat No. 516-0813) held in a Millipore filtration tower to avoid damaging the cells. The filters were washed in a 60 mm diameter plastic Petri dish with 10 ml of seawater. The dishes were scanned for characteristic *Meringosphaera* morphology (up to 13 undulating spines typically 16-25 μm long from a spherical body of 4-9 μm in cell diameter containing up to six green/yellow bodies and the cells are not motile) using a 40X objective of a Nikon Eclipse Ts2R inverted microscope, equipped with phase contrast. Single cells that appeared to contain green/yellow bodies were isolated using an Eppendorf TransferMan 4r micromanipulator and pulled glass pipettes. The cells were passed through droplets of minimal seawater to reduce contamination and frozen in 200μl PCR tubes with minimal seawater (e.g., <5 μl). Frozen single cells in PCR tubes were thawed and subjected to lysis and multiple displacement amplification (MDA) using the REPLI-g UltraFast Mini kit (Qiagen, Hilden, Germany) following the manufacturer’s instructions. The MDA samples were initially screened for the presence of *Meringosphaera* 18S rDNA with the centrohelid-specific primers Thx25F (5’-CATATGCTTGTCTCAAAGATTAAGCCA-3’) and Helio1979R (5’-CACACTTACWAGGAYTTCCTCGTTSAAGACG-3’) in a PCR reaction.^69^ After gel electrophoresis, if a band was present the PCR products were purified with ExoProStar 1-Step Kit (GE Healthcare; US77702) and Sanger-sequenced directly at Macrogen Europe. From this screening, 15 MDA samples containing *Meringosphaera* 18S rDNA were selected for the next steps.

#### Sequencing

The library preparations and sequencing were performed by SciLifeLab National Genomics Infrastructure (NGI), Stockholm, Sweden. Sequencing libraries were prepared with the TruSeq PCR-free library preparation, targeting an insert size of 350bp. Sequencing was performed in two batches: Batch 1 (became SAGs S1-4) were multiplexed on 1 lane and sequenced on Illumina NextSeq500 with paired-end ‘Mid-output’ chemistry; batch 2 (became SAGs S5-15) were multiplexed on 2 lanes and sequenced on Illumina NovaSeq6000 with paired-end ’NovaSeqStandard’ workflow in ’SP’ mode flowcell.

#### SAG assembly

The 15 datasets were trimmed using TrimGalore v0.6.1 (https://www.bioinformatics.babraham.ac.uk/projects/trim_galore/) with default parameters. Then normalised with bbnorm (bbmap v38.08^70^) with a minimum coverage value of 5 and a target value of 100 to help account for the biases introduced by MDA. The normalised reads were assembled into contigs with SPAdes (v3.13.1^71^) in careful mode (spades.py –careful -k auto). Basic parameters of the assemblies are listed in Table S2.

The contigs were made searchable as local databases with makeblastdb (blast 2.10.1+^72^) and searched with blastn. For the specific *Meringosphaera* 18S rDNA search GenBank accession MZ240752 was used as the query. For the general 18S rDNA search, a custom dataset containing diverse 18S rDNA sequences was used as the query (Figshare dataset D1.1). In the general search there were additional green algae picoeukaryote 18S rDNA identified in 2 of the SAGs (S5 and S7), but due to the lack of consistency, both across the samples and with the plastid identity, we believe this is contamination. For S7, we believe the degree of contamination was sufficient to increase the BUSCO completeness score (see Table S2).

We chose to use the 16S rDNA, *psbA* & *rbcL* genes as the plastid markers because they maximised the number of the SAGs with a plastid copy and are commonly sampled genes, meaning that environmental references could be included. For these plastid markers a custom dataset was used for each containing diverse plastid sequences (16S rDNA Figshare datasets D1.2, *psbA* D1.3, *rbcL* D1.4). The hits from the blast searches were aligned with MAFFT v7.407 (mafft -auto -adjustdirectionaccurately -reorder)^73^ and trimmed with trimAl v1.4.1^74^ (trimal -gappyout -fasta). For the most part, phylogenetic trees were made with IQ-TREE v1.6.5^75^ with ModelFinder^76^ to determine the best-fit model (for the 18S rDNA tree the TN+F+I+G model was chosen, for the plastid 16S rDNA the TIM3+F+I+G4 model, for psbA the GTR+F+G4 model and for rbcL the GTR+F+I+G4 model). Support values are from 1000 ultrafast bootstrap replicates and SH-aLRT test (iqtree -m TEST -bb 1000 -alrt 1000). However, the phylogenetic trees of the concatenated 18S and 28S rDNA (Figure 1A) sequences and the separate 28S rDNA phylogeny (Figshare D2.1B) were reconstructed with RAxML v. 8.2.12^77^ GTR models with 4 gamma categories and support values are from 1000 rapid bootstrap replicates.

The length of the *Meringosphaera* sequences used in the phylogenetic analyses are as follows: The *Meringosphaera* plastid 16S rDNA sequences (Figure 1B) were 1470-1480 bp in length. The *Meringosphaera* 18S rDNA sequences (Figshare D2.1A) were 1710-1720 bp in length. The complete *Meringosphaera psbA* sequences (Figure S1A) were 1059-1082 bp in length, but SAG S12 was incomplete and only 443 bp in length. The complete *Meringosphaera rbcL* sequences (Figure S1B) were 1458-1466 bp in length, but SAG S7 and S15 were incomplete and only 553 bp and 844 bp in length respectively. The *Meringosphaera* 28S rDNA sequences (Figshare D2.1B) were 2292 bp in length. The concatenated 18S and 28S rDNA sequences (for Figure 1A) were 3850 bp in length, and in order to get sufficient number of reference centrohelid 18S and 28S sequences from the same sample we used the long read OTU sequences generated by Jamy et al.^91^

#### Plastid genome assembly & comparisons

The plastid genomes were predicted and assembled with GetOrganelle v1.7.3.3^78^ with the other_pt database (get_organelle_from_- reads.py -F other_pt -R 15 -k 21,45,65,85,105). In addition, GetOrganelle was performed with the seed option (-s) using the identified SAG S4 plastid genome to assist with plastid identification in the other SAGs. Six plastid genomes were assembled as circular by GetOrganelle and are predicted as ‘complete’ (S2, S3, S4, S6, S8, and S14). We did not verify them with PCR. Plastid contigs were annotated with MFannot (https://megasun.bch.umontreal.ca/cgi-bin/mfannot/mfannotInterface.pl) and were drawn with OG-Draw.^79^ The MFannot predictions of absent plastid genes within *Meringosphaera* plastids were confirmed by manual verification using Blast. For the plastid genome comparisons, the annotated SAG plastid genomes were compared to the four published photosynthetic Dictyochophyceae plastid genomes available at the time (GenBank accession numbers: MK518352, MK518353, MK561359 & MK561360). The analysis was conducted and visualised with R v.4.2.0^80^ in RStudio.^81^

#### Co-assemblies & host-encoded plastid associated genes

The MDA process is known to produce uneven coverage that can lead to errors in assembly, meaning that gene absences at the SAG level could be either biological or an artefact. To help address this, and to increase the coverage overall, we formed co-assemblies for the investigations of the host genes. First, the plastid sequences were removed from the SAGS, and the remaining reads were clustered based on >99% *Meringosphaera* 18S rDNA. Within these clusters the reads were co-assembled in the same way as described above for the single assemblies. This created three co-assemblies: COSAG1 (from SAG S2,3 & 6), COSAG2a (SAG S10,13), & COSAG2b (SAG S1,4,5,7,8,9,11,12,14,15). In this way, the co-assemblies included SAGs both with and without plastids. The completeness of the SAGs and COSAGS was assessed with BUSCO v5.3.1^82^ in genome mode with the eukaryote database of 255 markers (Table S2). Basic parameters of the co-assemblies are listed in Table S2.

Open reading frames (ORFs) and amino acid sequences were predicted with Prodigal v2.6.3^83^ using default parameters. The amino acid sequences were made searchable as local blast databases (as above). These were searched for the 31 target proteins using reference databases across diverse taxa as the queries (databases from Schön et al.^57^: Figshare Collection. https://doi.org/10.6084/m9.figshare.c.5388176.v3, list of proteins also based on Hehenberger et al.^21^). In addition, we also searched for 35 metabolic transporters found to be kleptoplast-targeted in the euglenozoan *Rapaza viridis*.^25^The resulting homologs were aligned with the reference database and IQ trees were made according to the method described above. The trees were manually inspected to identify any homologs that grouped with known plastid-bearing species. The identity of the up- and downstream proteins surrounding these candidates were then assessed. The surrounding proteins were run through three different databases: Blastp tsa_nr mode (Transcriptome Shotgun Assembly proteins), Blastp nr mode (non-redundant protein sequences), and SequenceServer 2.0.0^84^ with the EukProt V3 database^92^ selected taxonomic groups Centroplashelida, Haptophyta and Dictyochophyceae. The top 20 hits were taken from each of these searches and IQ trees were made of the alignments; from these trees the identity of the contigs was established. Only candidates whose up- and downstream proteins had possible centrohelid identity were kept. Coverage was checked manually across the candidate contigs to check for mis-assemblies, using BWA v0.7.8^85^ and visualised with IGV v2.4.2.^86^ Each candidate was manually checked for completeness, using alignments to a variety of homologs, and particular care was taken with the N and C termini. The completeness of each candidate is noted in Table S3, along with a general summary of each candidate. Only if the N-terminus was complete did we predict the subcellular localisation with DEEPLOC 2.0^87^ and TargetP 2.0.^88^ For the transporters, only candidates with predicted plastid-targeting were kept because often homologous transporters function in different cellular compartments. Candidate proteins that had been split by Prodigal due to the presence of introns were concatenated together manually and when this was necessary it is noted in Table S3.

In addition, we searched for the proteins encoded by genes identified as plastid-to-nucleus transferred within a Dictyochophyceae species.^49^ Homologs of these nine proteins (*acpP, ilvB, petF, psb28, rpl12, rpl32, syfB, ycf35* and *ycf42*) were not found in any of the *Meringosphaera* co-assemblies. However, some of these were still within the plastid genomes: in particular, *acpP* was found in the group 1 plastids, and *rpl12* and *rpl32* were found in all of the SAG plastids (see Figures 4A and S4).

#### Card-Fish

Water samples (0.5L) were filtered on 5μm 47mm-diameter polycarbonate hydrophilic membrane filters (Whatman) by gravity filtration. The filters were fixed in the dark at room temperature in 2ml of 4% paraformaldehyde for 1 hour. The filters were then rinsed three times with 0.2μm-filtered sea water and stored frozen at -20°C.

The CARD-FISH procedure was performed on these filters at a later date (a few months after collection). First, the filters were embedded in 0.2% low melting point agarose. Next, an initial permeabilization step of incubation in 10mg ml^-1^ lysozyme solution for 1 hour at 35°C, followed by a MilliQ wash. The rest of the procedure followed the protocol provided by Piwosz et al.^93^ Briefly, the filters were then moved to 0.01M HCL solution for a 20-minute incubation at room temperature, before being washed in PBS and DI water. Hybridisation occurred at 35°C for ~20 hours, for which the 50ng/μl probe solution was mixed with the appropriate hybridisation buffer (see Piwosz et al.^93^) in a 1:99 dilution, producing a final probe concentration of 0.5ng/μl. The formamide percentage in the hybridisation buffer was optimised per probe, and the final percentage used is given in Table S4. The filters were then washed at 37°C for 30 minutes, and the concentration of NaCl in the wash buffer was determined by the formamide percentage of the hybridisation buffer. The filters were incubated in 0.01% PBS-Triton for 45 minutes at 37°C. They were then incubated at 37°C in the dark with the amplification buffer and fluorochrome-labelled tyramide solution for the CARD step. Alexa fluor 488 was used in this process. The filters were then incubated in 0.01% PBS-Triton for 15 minutes in the dark at 37°C. The final wash occurred in MilliQ, and lastly ethanol (100%). The filters were then air-dried. The filters were mounted onto slides with 1μg ml^-1^ DAPI in ProLong™ Diamond Antifade mountant (Invitrogen™). The slides were dried for 24 hours in the dark at room temperature and stored frozen at -20°C until visualization with a confocal microscope.

The CARD-FISH probes used in this experiment are listed in Table S4. One of which was designed for this project, Mer482, and it targets both group 1 and 2 *Meringosphaera* 18S rRNA (though within the April 2021 CARD-FISH samples only group 2 hosts are present). Mer482 probe was designed with the help of oligoN-design v0.1.0 pipeline^94^ and then was checked for specificity with the Silva 18S rRNA Arb database.^95^ Probe function was predicted by MathFISH,^96,97^and the predicted formamide concentration was used as the starting point.

Each sample filter was cut into 16 equal slices (of ~1.1cm^2^) in order that the controls could be applied to the same sample. In this way, every CARD-FISH experiment included both a negative and positive control. In the negative control the Mer482 specific HRP probe for *Meringosphaera* was absent but the Alexa 488 dye was added to check for non-specific binding. In the positive control a general eukaryote probe EUK1209R (EUK1195) was used instead of the *Meringosphaera* probe so to ensure that the CARD-FISH procedure worked optimally.

Images were taken on inverted confocal microscope Zeiss LSM 780 (Zeiss Berlin Germany) using the 405, 488 and 633nm lasers with the 63×/1.4 oil objective lens. The ZEN black 2011 software was used to control the system and process the images. The same gain and intensity per channel were used when comparing between all the sample conditions and across the controls.

#### Environmental monitoring with qPCR

Surface water samples (0.5-1L) were filtered onto a 47mm diameter 5μm filter (Whatman) by gravity filtration or onto a 25mm diameter 5μm filter (Whatman) held in 25-mm diameter Swinnex filter holder (Millipore Billerca MA USA) using a peristaltic pump (Cole-Parmer, Vernon Hills, IL USA). We aimed to filter 1L of water per replicate and to have 3 replicates per sample location when possible. There were some differences in which of the three locations were sampled per month, this information can be seen in Figures S2 that splits the qPCR data by sampling location. The filters were snap frozen in liquid nitrogen and stored at -80°C. DNA was extracted from the filters with the DNeasy Plant kit (Qiagen, Hilden, Germany) with the following modifications to the manufacturer’s protocol: Tubes containing the 400μl API buffer and the filter were subject to three freeze/thaw cycles (alternating from submerged in liquid nitrogen to 65°C). Glass beads (0.65g of a 50:50 mix of 2.8mm and 1.4mm Zirconium oxide beads (Precellys)) were added to the tubes, and then these were subjected to a 2-minute bead beat step (at 8500 rpm). 45μl Proteinase K was added and the samples were incubated for 1 hour at 55°C. The rest of the procedure followed the Qiagen protocol starting with the addition of RNase A. The final elution volume was 30μl. Extracted DNA was stored at -20°C.

qPCR was performed using the TaqMan chemistry (Applied Biosystems) and using a Step One Plus Real-Time PCR system (Applied Biosystems). The qPCR program was: 50°C for 2 min, 95°C for 10 min and 45 cycles of 95°C for 15 secs followed by the Tm temperature for 1 min (the Tm temperatures per probe are listed in Table S4). The reaction volume was 25μl per well, containing: 12.5μl 2X TaqMan buffer (Applied Biosystems), 8μl nuclease-free water, 0.4μmol L^-1^ forward primer, 0.4μmol L^-1^ reverse primer, 0.2μmol L^-1^ probe & 2μl of template DNA). The target-specific TaqMAN probe was 5’ labelled with a fluorescent reporter FAM (6-carboxyfluoesceom) or VIC (2′-chloro-7′phenyl-1,4-dichloro-6-carboxy-fluorescein) (see Table S4 for details per probe) and 3’labelled with TAMRA (6-caroboxytetramethylrhomadine) as a quenching dye. On every plate, standards for an 8-point standard curve were included and were run in duplicates. The standard curve was made from a 10-fold dilution series ranging from 10^8^ to 10^1^ gene copies per reaction using synthesized Gblocks (IDT) of the target region. Every plate also included 3 wells for the negative controls, where nuclease-free water was used instead of template DNA. Each sample was run with 2 or 3 technical replicates. If the target could not be detected in all of the technical replicates the sample was marked as detected but not quantifiable and were plotted with a value of 1.1. Gene copy numbers were calculated from the mean cycle threshold (Ct) value of the technical replicates, this value was then quantified into copy number using the standard curve.

The qPCR primers and probes in this study were designed using the Primer Express (Applied Biosystems) software, and their specificity was tested against the Silva database, NCBI, and with the alignments created from the sequencing results. Cross-reactivity between the primers/probes of the different groups were tested, e.g., the 18S group 1 was tested on group 2 standards, and no amplification occurred. The details of the primers and probes are given in Table S4.

### Quantification And Statistical Analysis

The statistical analysis was conducted with R v.4.2.0^80^ in RStudio.^81^ Within the qPCR results, the abundance of *Meringosphaera* group 2 was analysed by ANOVA with month as a factor (N = 57). The data was tested for normality by creating Q-Q and residual vs fitted values plots in R. When error bars are shown in a figure, the type of error used is noted in the legend.

## Supplementary Material

Movie S1

Supplementary Material

Table S3

## Data Availability

The identified genetic sequences have been deposited at GenBank and are publicly available as of the date of publication (*Meringosphaera* 18S rDNA sequences GenBank:OQ075975 to OQ075989, *Meringosphaera* 28S rDNA sequences GenBank: OR195151 to OR195157 and OR196762 to OR196769, *Meringosphaera* plastid 16S rDNA sequences GenBank: OQ091774 to OQ091781, plastid *psbA* sequences GenBank: OQ078560 to OQ078568, plastid *rbcL* sequences GenBank: OQ078569 to OQ078579, and the complete plastid genomes GenBank: OQ161668 to OQ161673). The raw reads data have been deposited at NCBI Sequence Read Archive: BioProject PRJNA917255, accession numbers: SAMN32532880 to SAMN32532894. All data files are available at Figshare https://doi.org/10.6084/m9.figshare.c.6313464, this includes all the plastid contigs (both complete and incomplete), the host-encoded plastid-associated protein candidates, single gene trees, the assembled reads of the SAGs and COSAGS, and the qPCR results. All custom scripts used in this study are publicly available at GitHub: https://github.com/MeganSorensen/Meringosphaera_SAGs. Any additional information required to reanalyse the data reported in this paper is available from the lead contact upon request.

## Highlights

- *Meringosphaera* protists engage in kleptoplastidic behavior
- The kleptoplasts are derived from dictyochophyte algae
- Seasonal dynamics of hosts and plastids differ, suggesting plastid switching
- Host nuclear genomes encode possible kleptoplast-targeted genes

## References

[1] Sibbald SJ, Archibald JM (2020). Genomic insights into plastid evolution. Genome Biol Evol.

[2] Adl SM, Simpson AGB, Farmer MA, Andersen RA, Anderson OR, Barta JR, Bowser SS, Brugerolle G, Fensome RA, Fredericq S (2005). The new higher level classification of eukaryotes with emphasis on the taxonomy of protists. J Eukaryot Microbiol.

[3] Archibald JM (2009). The puzzle of plastid evolution. Curr Biol.

[4] Cavalier-Smith T (1982). The origins of plastids. Biol J Linn Soc.

[5] Keeling PJ (2010). The endosymbiotic origin, diversification and fate of plastids. Philos Trans R Soc Lond B Biol Sci.

[6] Ponce-Toledo RI, López-García P, Moreira D (2019). Horizontal and endosymbiotic gene transfer in early plastid evolution. New Phytol.

[7] Hinde R (1983). Algal Symbiosis A Continuum of Interaction Strategies.

[8] Pelletreau KN, Bhattacharya D, Price DC, Worful JM, Moustafa A, Rumpho ME (2011). Sea slug kleptoplasty and plastid maintenance in a metazoan. Plant Physiol.

[9] Van Steenkiste NWL, Stephenson I, Herranz M, Husnik F, Keeling PJ, Leander BS (2019). A new case of kleptoplasty in animals: marine flatworms steal functional plastids from diatoms. Sci Adv.

[10] Jesus B, Jauffrais T, Trampe ECL, Goessling JW, Lekieffre C, Meibom A, Kühl M, Geslin E (2022). Kleptoplast distribution, photosynthetic efficiency and sequestration mechanisms in intertidal benthic foraminifera. ISME J.

[11] Gustafson DE, Stoecker DK, Johnson MD, Van Heukelem WF, Sneider K (2000). Cryptophyte algae are robbed of their organelles by the marine ciliate Mesodinium rubrum. Nature.

[12] Johnson MD (2011). Acquired phototrophy in ciliates: a review of cellular interactions and structural adaptations. J Eukaryot Microbiol.

[13] Waller RF, Koŕený L, Hirakawa Y (2017). Advances in Botanical Research Secondary Endosymbioses.

[14] Stoecker DK, Johnson MD, de Vargas C, Not F (2009). Acquired phototrophy in aquatic protists. Aquat Microb Ecol.

[15] Bodył A (2018). Did some red alga-derived plastids evolve via kleptoplastidy? A hypothesis. Biol Rev Camb Philos Soc.

[16] Martin W, Herrmann RG (1998). Gene transfer from organelles to the nucleus: how much, what happens, and why?1. Plant Physiol.

[17] Martin W, Rujan T, Richly E, Hansen A, Cornelsen S, Lins T, Leister D, Stoebe B, Hasegawa M, Penny D (2002). Evolutionary analysis of Arabidopsis, cyanobacterial, and chloroplast genomes reveals plastid phylogeny and thousands of cyanobacterial genes in the nucleus. Proc Natl Acad Sci USA.

[18] Qiu H, Price DC, Weber APM, Facchinelli F, Yoon HS, Bhattacharya D (2013). Assessing the bacterial contribution to the plastid proteome. Trends Plant Sci.

[19] Johnson MD (2011). The acquisition of phototrophy: adaptive strategies of hosting endosymbionts and organelles. Photosynth Res.

[20] Gast RJ, Moran DM, Dennett MR, Caron DA (2007). Kleptoplasty in an Antarctic dinoflagellate: caught in evolutionary transition?. Environ Microbiol.

[21] Hehenberger E, Gast RJ, Keeling PJ (2019). A kleptoplastidic dinoflagellate and the tipping point between transient and fully integrated plastid endosymbiosis. Proc Natl Acad Sci USA.

[22] Husnik F, Nikoh N, Koga R, Ross L, Duncan RP, Fujie M, Tanaka M, Satoh N, Bachtrog D, Wilson ACC (2013). Horizontal gene transfer from diverse bacteria to an insect genome enables a tripartite nested mealybug symbiosis. Cell.

[23] Wardell GE, Hynes MF, Young PJ, Harrison E (2022). Why are rhizobial symbiosis genes mobile?. Philos Trans R Soc Lond B Biol Sci.

[24] Wisecaver JH, Hackett JD (2010). Transcriptome analysis reveals nuclear-encoded proteins for the maintenance of temporary plastids in the dinoflagellate Dinophysis acuminata. BMC Genomics.

[25] Karnkowska A, Yubuki N, Maruyama M, Yamaguchi A, Kashiyama Y, Suzaki T, Keeling PJ, Hampl V, Leander BS (2023). Euglenozoan kleptoplasty illuminates the early evolution of photoendosymbiosis. Proc Natl Acad Sci USA.

[26] Donohoo SA, Wade RM, Sherwood AR (2020). Finding the sweet spot: sub-ambient light increases fitness and kleptoplast survival in the sea slug *Plakobranchus* cf. *ianthobaptus* Gould, 1852. Biol Bull.

[27] Cartaxana P, Morelli L, Quintaneiro C, Calado G, Calado R, Cruz S (2018). Kleptoplast photoacclimation state modulates the photobehaviour of the solar-powered sea slug Elysia viridis. J Exp Biol.

[28] Torres JP, Lin Z, Winter JM, Krug PJ, Schmidt EW (2020). Animal biosynthesis of complex polyketides in a photosynthetic partnership. Nat Commun.

[29] Gallop A, Bartrop J, Smith DC (1980). The biology of chloroplast acquisition by Elysia viridis. Proc R Soc Lond B.

[30] Jauffrais T, LeKieffre C, Koho KA, Tsuchiya M, Schweizer M, Bernhard JM, Meibom A, Geslin E (2018). Ultrastructure and distribution of kleptoplasts in benthic foraminifera from shallow-water (photic) habitats. Mar Micropaleontol.

[31] Johnson MD, Oldach D, Delwiche CF, Stoecker DK (2007). Retention of transcriptionally active cryptophyte nuclei by the ciliate Myrionecta rubra. Nature.

[32] Onuma R, Horiguchi T (2015). Kleptochloroplast enlargement, karyoklepty and the distribution of the cryptomonad nucleus in Nusuttodinium (= Gymnodinium) aeruginosum (Dinophyceae). Protist.

[33] Larkum AWD, Lockhart PJ, Howe CJ (2007). Shopping for plastids. Trends Plant Sci.

[34] Husnik F, Tashyreva D, Boscaro V, George EE, Lukeš J, Keeling PJ (2021). Bacterial and archaeal symbioses with protists. Curr Biol.

[35] Keeling PJ (2013). The number, speed, and impact of plastid endosym-bioses in eukaryotic evolution. Annu Rev Plant Biol.

[36] Singer A, Poschmann G, Mühlich C, Valadez-Cano C, Hänsch S, Hüren V, Rensing SA, Stühler K, Nowack ECM (2017). Massive protein import into the early-evolutionary-stage photosynthetic organelle of the amoeba Paulinella chromatophora. Curr Biol.

[37] Georgiev AA, Georgieva ML, Gololobova MA (2021). New observations of meringosphaera Mediterranea from Russian Arctic seas, including a review of global distribution. Hoboctκ CκcτeMaτκικ Hk3zkh PacTeHNN.

[38] Leadbeater BSC (1974). Ultrastructural observations on nanoplankton collected from the coast of Jugoslavia and the Bay of Algiers. J Mar Biol Assoc UK.

[39] LeRoi J-M, Hallegraeff GM (2006). Scale-bearing nanoflagellates from southern Tasmanian coastal waters, Australia. II. Species of Chrysophyceae (Chrysophyta), Prymnesiophyceae (Haptophyta, excluding Chrysochromulina) and Prasinophyceae (Chlorophyta). Botanica Marina.

[40] Thorrington-Smith M (1970). Some new and little known phytoplankton forms from the West Indian Ocean. Br Phycol J.

[41] Obiol A, Muhovic I, Massana R (2021). Oceanic heterotrophic flagellates are dominated by a few widespread taxa. Limnol Oceanogr.

[42] Lohmann HT (1902). Wissenschaftl Meeresontersuchung herausgeg v d Kommiss zur Untersuch, d deutschen Meere in Kiel und d Biol Anstalt auf Helgoland.

[43] Norris RE (1970). Extant siliceous microalgae from the Indian Ocean.

[44] Zlatogursky VV, Shɨshkin Y, Drachko D, Burki F (2021). The longtime orphan protist Meringosphaera mediterranea Lohmann, 1902 [1903] is a centrohelid heliozoan. J Eukaryot Microbiol.

[45] Mikrjukov KA, Siemensma FJ, Patterson DJ (2000). The Illustrated Guide to the Protozoa.

[46] Patterson DJ, Dürrschmidt M (1987). Selective retention of chloroplasts by algivorous heliozoa: fortuitous chloroplast symbiosis?. Eur J Protistol.

[47] Matzke B, Schwarzmeier E, Loos E (1990). Maltose excretion by the symbiotic Chlorella of the heliozoan Acanthocystis turfacea. Planta.

[48] Burki F, Kaplan M, Tikhonenkov DV, Zlatogursky V, Minh BQ, Radaykina LV, Smirnov A, Mylnikov AP, Keeling PJ (2016). Untangling the early diversification of eukaryotes: a phylogenomic study of the evolutionary origins of Centrohelida, Haptophyta and Cryptista. Proc Biol Sci.

[49] Han KY, Maciszewski K, Graf L, Yang JH, Andersen RA, Karnkowska A, Yoon HS (2019). Dictyochophyceae plastid genomes reveal unusual variability in their organization. J Phycol.

[50] Janouškovec J, Horák A, Oborník M, Lukeš J, Keeling PJ (2010). A common red algal origin of the apicomplexan, dinoflagellate, and heterokont plastids. Proc Natl Acad Sci USA.

[51] Ong HC, Wilhelm SW, Gobler CJ, Bullerjahn G, Jacobs MA, McKay J, Sims EH, Gillett WG, Zhou Y, Haugen E (2010). Analyses of the complete chloroplast genome sequences of two members of the Pelagophyceae: Aureococcus Anophagefferens Ccmp1984 and Aureoumbra lagunensis Ccmp15071. J Phycol.

[52] Kayama M, Maciszewski K, Yabuki A, Miyashita H, Karnkowska A, Kamikawa R (2020). Highly reduced plastid genomes of the non-photosynthetic Dictyochophyceans Pteridomonas spp. (Ochrophyta, SAR) are retained for tRNA-Glu-based organellar heme biosynthesis. Front Plant Sci.

[53] de Koning AP, Keeling PJ (2004). Nucleus-encoded genes for plastid-targeted proteins in Helicosporidium: functional diversity of a cryptic plastid in a parasitic alga. Eukaryot Cell.

[54] Ševčíková T, Yurchenko T, Fawley KP, Amaral R, Strnad H, Santos LMA, Fawley MW, Eliáš M (2019). Plastid genomes and proteins illuminate the evolution of eustigmatophyte algae and their bacterial endosymbionts. Genome Biol Evol.

[55] Zhang M, Chen N (2022). Comparative analysis of Thalassionema chloroplast genomes revealed hidden biodiversity. BMC Genomics.

[56] Kovács-Bogdán E, Benz JP, Soll J, Bölter B (2011). Tic20 forms a channel independent of Tic110 in chloroplasts. BMC Plant Biol.

[57] Schön ME, Zlatogursky VV, Singh RP, Poirier C, Wilken S, Mathur V, Strassert JFH, Pinhassi J, Worden AZ, Keeling PJ (2021). Single cell genomics reveals plastid-lacking Picozoa are close relatives of red algae. Nat Commun.

[58] Przybyla-Toscano J, Roland M, Gaymard F, Couturier J, Rouhier N (2018). Roles and maturation of iron–sulfur proteins in plastids. J Biol Inorg Chem.

[59] Nishitani G, Nagai S, Hayakawa S, Kosaka Y, Sakurada K, Kamiyama T, Gojobori T (2012). Multiple plastids collected by the dinoflagellate *Dinophysis mitra* through kleptoplastidy. Appl Environ Microbiol.

[60] Schweikert M, Elbraächter M (2004). First ultrastructural investigations of the consortium between a phototrophic eukaryotic endocytobiont and Podolampas bipes (Dinophyceae). Phycologia.

[61] Ikäavalko J, Gradinger R (1997). Flagellates and heliozoans in the Greenland Sea ice studied alive using light microscopy. Polar Biol.

[62] Vørs N, Buck KR, Chavez FP, Eikrem W, Hansen LE, Østergaard JB, Thomsen HA (1995). Nanoplankton of the equatorial Pacific with emphasis on the heterotrophic protists. Deep Sea Res II.

[63] Wille N (1909). Die natürlichen Pflanzenfamilien, Nachträge zum I Teil, Abteilung 2 über die Jahre 1890 bis 1910.

[64] Maeda T, Hirose E, Chikaraishi Y, Kawato M, Takishita K, Yoshida T, Verbruggen H, Tanaka J, Shimamura S, Takaki Y (2012). Algivore or phototroph? Plakobranchus ocellatus (Gastropoda) continuously acquires kleptoplasts and nutrition from multiple algal species in nature. PLoS One.

[65] Nishitani G, Yamaguchi M (2018). Seasonal succession of ciliate Mesodinium spp. with red, green, or mixed plastids and their association with cryptophyte prey. Sci Rep.

[66] Decelle J, Probert I, Bittner L, Desdevises Y, Colin S, de Vargas C, Galí M, Simó R, Not F (2012). An original mode of symbiosis in open ocean plankton. Proc Natl Acad Sci USA.

[67] Binga EK, Lasken RS, Neufeld JD (2008). Something from (almost) nothing: the impact of multiple displacement amplification on microbial ecology. ISME J.

[68] Gornik SG, Febrimarsa, Cassin AM, MacRae JI, Ramaprasad A, Rchiad Z, McConville MJ, Bacic A, McFadden GI, Pain A (2015). Endosymbiosis undone by stepwise elimination of the plastid in a parasitic dinoflagellate. Proc Natl Acad Sci.

[69] Cavalier-Smith T, von der Heyden S (2007). Molecular phylogeny, scale evolution and taxonomy of centrohelid heliozoa. Mol Phylogenet Evol.

[70] Bushnell B (2014). BBMap: a fast, accurate, splice-aware aligner.

[71] Bankevich A, Nurk S, Antipov D, Gurevich AA, Dvorkin M, Kulikov AS, Lesin VM, Nikolenko SI, Pham S, Prjibelski AD (2012). SPAdes: a new genome assembly algorithm and its applications to single-cell sequencing. J Comput Biol.

[72] Sayers EW, Bolton EE, Brister JR, Canese K, Chan J, Comeau DC, Connor R, Funk K, Kelly C, Kim S (2022). Database resources of the national center for biotechnology information. Nucleic Acids Res.

[73] Katoh K, Standley DM (2013). MAFFT Multiple Sequence Alignment, software version 7: improvements in performance and usability. Mol Biol Evol.

[74] Capella-Gutiérrez S, Silla-Martínez JM, Gabaldón T (2009). trimAl: a tool for automated alignment trimming in large-scale phylogenetic analyses. Bioinformatics.

[75] Minh BQ, Schmidt HA, Chernomor O, Schrempf D, Woodhams MD, von Haeseler A, Lanfear R (2020). IQ-TREE 2: new models and efficient methods for phylogenetic inference in the genomic era. Mol Biol Evol.

[76] Kalyaanamoorthy S, Minh BQ, Wong TKF, von Haeseler A, Jermiin LS (2017). ModelFinder: fast model selection for accurate phylogenetic estimates. Nat Methods.

[77] Stamatakis A (2014). RAxML version 8: a tool for phylogenetic analysis and post-analysis of large phylogenies. Bioinformatics.

[78] Jin JJ, Yu WB, Yang JB, Song Y, dePamphilis CW, Yi TS, Li DZ (2020). GetOrganelle: a fast and versatile toolkit for accurate de novo assembly of organelle genomes. Genome Biol.

[79] Greiner S, Lehwark P, Bock R (2019). OrganellarGenomeDRAW (OGDRAW), version 1.3.1: expanded toolkit for the graphical visualization of organellar genomes. Nucleic Acids Res.

[80] R Core Team (2020). R: A Language and Environment for Statistical Computing.

[81] RStudio Team (2019). RStudio: Integrated Development for R.

[82] Simao FA, Waterhouse RM, Ioannidis P, Kriventseva EV, Zdobnov EM (2015). BUSCO: assessing genome assembly and annotation completeness with single-copy orthologs. Bioinformatics.

[83] Hyatt D, Chen GL, LoCascio PF, Land ML, Larimer FW, Hauser LJ (2010). Prodigal: prokaryotic gene recognition and translation initiation site identification. BMC Bioinformatics.

[84] Priyam A, Woodcroft BJ, Rai V, Moghul I, Munagala A, Ter F, Chowdhary H, Pieniak I, Maynard LJ, Gibbins MA (2019). Sequenceserver: a modern graphical user interface for custom BLAST databases. Mol Biol Evol.

[85] Li H, Durbin R (2009). Fast and accurate short read alignment with Burrows-Wheeler transform. Bioinformatics.

[86] Thorvaldsdóttir H, Robinson JT, Mesirov JP (2013). Integrative Genomics Viewer (IGV): high-performance genomics data visualization and exploration. Brief Bioinform.

[87] Thumuluri V, Almagro Armenteros JJ, Johansen AR, Nielsen H, Winther O (2022). DeepLoc 2.0: multi-label subcellular localization prediction using protein language models. Nucleic Acids Res.

[88] Almagro Armenteros JJ, Salvatore M, Emanuelsson O, Winther O, von Heijne G, Elofsson A, Nielsen H (2019). Detecting sequence signals in targeting peptides using deep learning. Life Sci Alliance.

[89] Vernette C, Lecubin J, Sánchez P, Sunagawa S, Delmont TO, Acinas SG, Pelletier E, Hingamp P, Lescot M, Tara Oceans Coordinators (2022). The Ocean Gene Atlas v2.0: online exploration of the biogeography and phylogeny of plankton genes. Nucleic Acids Res.

[90] Villar E, Vannier T, Vernette C, Lescot M, Cuenca M, Alexandre A, Bachelerie P, Rosnet T, Pelletier E, Sunagawa S (2018). The Ocean Gene Atlas: exploring the biogeography of plankton genes online. Nucleic Acids Res.

[91] Jamy M, Biwer C, Vaulot D, Obiol A, Jing H, Peura S, Massana R, Burki F (2022). Global patterns and rates of habitat transitions across the eukaryotic tree of life. Nat Ecol Evol.

[92] Richter DJ, Berney C, Strassert JFH, Poh Y-P, Herman EK, Muñoz-Gómez SA, Wideman JG, Burki F, de Vargas C (2020). EukProt: a database of genome-scale predicted proteins across the diversity of eukaryotes. Peer Community Journal.

[93] Piwosz K, Mukherjee I, Salcher MM, Grujčić V, Šimek K (2021). CARD-FISH in the sequencing era: opening a new universe of protistan ecology. Front Microbiol.

[94] Sandin M, Walde M oligoN-design v0.1.0: a pipeline for the high throughput design of specific primers/probes Version v0.1.0.

[95] Quast C, Pruesse E, Yilmaz P, Gerken J, Schweer T, Yarza P, Peplies J, Glöckner FO (2013). The SILVA ribosomal RNA gene database project: improved data processing and webbased tools. Nucleic Acids Res.

[96] Yilmaz LS, Parnerkar S, Noguera DR (2011). mathFISH, a web tool that uses thermodynamics-based mathematical models for in silico evaluation of oligonucleotide probes for fluorescence in situ hybridization. Appl Environ Microbiol.

[97] Yilmaz LS, Noguera DR (2007). Development of thermodynamic models for simulating probe dissociation profiles in fluorescence in situ hybridization. Biotechnol Bioeng.

