# Supplementary material for: A novel kleptoplastidic symbiosis revealed in the marine centrohelid Meringosphaera with evidence of genetic integration
Source: Curr Biol. Author manuscript; Available in PMC 2023 Sep 11. (PMC7615077; doi:10.1016/j.cub.2023.07.017)
Supplement: Supplementary Material [file EMS186826-supplement-Supplementary_Material.pdf]

Current Biology, Volume *33*

Supplemental Information

A novel kleptoplastidic symbiosis revealed in the marine centrohelid *Meringosphaera* with evidence of genetic integration

Megan E.S. Sørensen, Vasily V. Zlatogursky, Ioana Onuţ-Brännström, Anne Walraven, Rachel A. Foster, and Fabien Burki

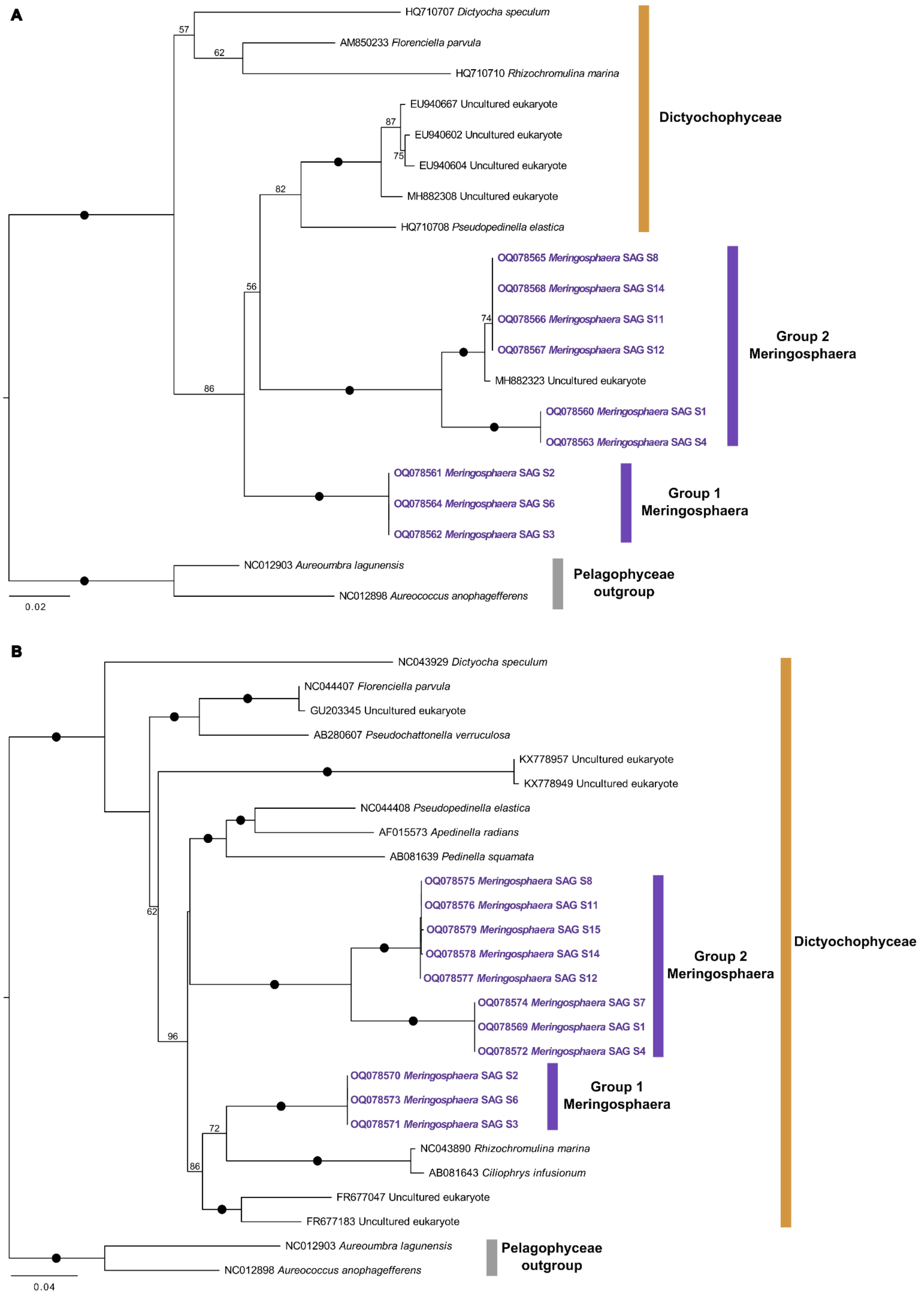
A
HQ710707 Dictyocha speculum
AM850233 Florenciella parvula
HQ710710 Rhizochromulina marina
EU940667 Uncultured eukaryote
EU940602 Uncultured eukaryote
EU940604 Uncultured eukaryote
MH882308 Uncultured eukaryote
HQ710708 Pseudopedinella elastica
OQ078565 Meringosphaera SAG S8
OQ078568 Meringosphaera SAG S14
OQ078566 Meringosphaera SAG S11
OQ078567 Meringosphaera SAG S12
MH882323 Uncultured eukaryote
OQ078560 Meringosphaera SAG S1
OQ078563 Meringosphaera SAG S4
OQ078561 Meringosphaera SAG S2
OQ078564 Meringosphaera SAG S6
OQ078562 Meringosphaera SAG S3
NC012903 Aureoumbra lagunensis
NC012898 Aureococcus anophagefferens
57
62
87
75
82
56
74
86
0.02
Dictyochophyceae
Group 2 Meringosphaera
Group 1 Meringosphaera
Pelagophyceae outgroup
B
NC043929 Dictyocha speculum
NC044407 Florenciella parvula
GU203345 Uncultured eukaryote
AB280607 Pseudochattonella verruculosa
KX778957 Uncultured eukaryote
KX778949 Uncultured eukaryote
NC044408 Pseudopedinella elastica
AF015573 Apedinella radians
AB081639 Pedinella squamata
OQ078575 Meringosphaera SAG S8
OQ078576 Meringosphaera SAG S11
OQ078579 Meringosphaera SAG S15
OQ078578 Meringosphaera SAG S14
OQ078577 Meringosphaera SAG S12
OQ078574 Meringosphaera SAG S7
OQ078569 Meringosphaera SAG S1
OQ078572 Meringosphaera SAG S4
OQ078570 Meringosphaera SAG S2
OQ078573 Meringosphaera SAG S6
OQ078571 Meringosphaera SAG S3
NC043890 Rhizochromulina marina
AB081643 Ciliophrys infusionum
FR677047 Uncultured eukaryote
FR677183 Uncultured eukaryote
NC012903 Aureoumbra lagunensis
NC012898 Aureococcus anophagefferens
62
96
72
86
0.04
Group 2 Meringosphaera
Group 1 Meringosphaera
Dictyochophyceae
Pelagophyceae outgroup

Figure S1. Diversity and phylogeny of *psbA* and *rbcL* from the *Meringosphaera* plastid sequences, related to Figure 1. A) Maximum likelihood tree of selected cultured and representative *psbA* sequences showing the *Meringosphaera* plastids sister to the Dictyochophyceae and split into two distinct groups that correspond to the groups in the 18S and 16S rDNA phylogenies (Figure 1 and Figshare repository D2.1A). The tree was reconstructed with a GTR+F+G4 model, chosen by ModelFinder. **B)** Maximum likelihood tree of selected cultured and representative *rbcL* sequences showing the *Meringosphaera* plastid sequences among the Dictyochophyceae and split into two distinct groups that correspond to the groups in the 18S and 16S rDNA phylogenies (Figure 1 and Figshare repository D2.1A). The tree was reconstructed with a GTR+F+I+G4 model, chosen by ModelFinder. For both panels, Pelagophyceae sequences were used as the outgroup, and support values correspond to ultrafast bootstrap values from 1000 replicates. Support values over 50% are shown on the tree, with values over 90% represented by a black circle on the branch. See Methods for details regarding the tree formation.

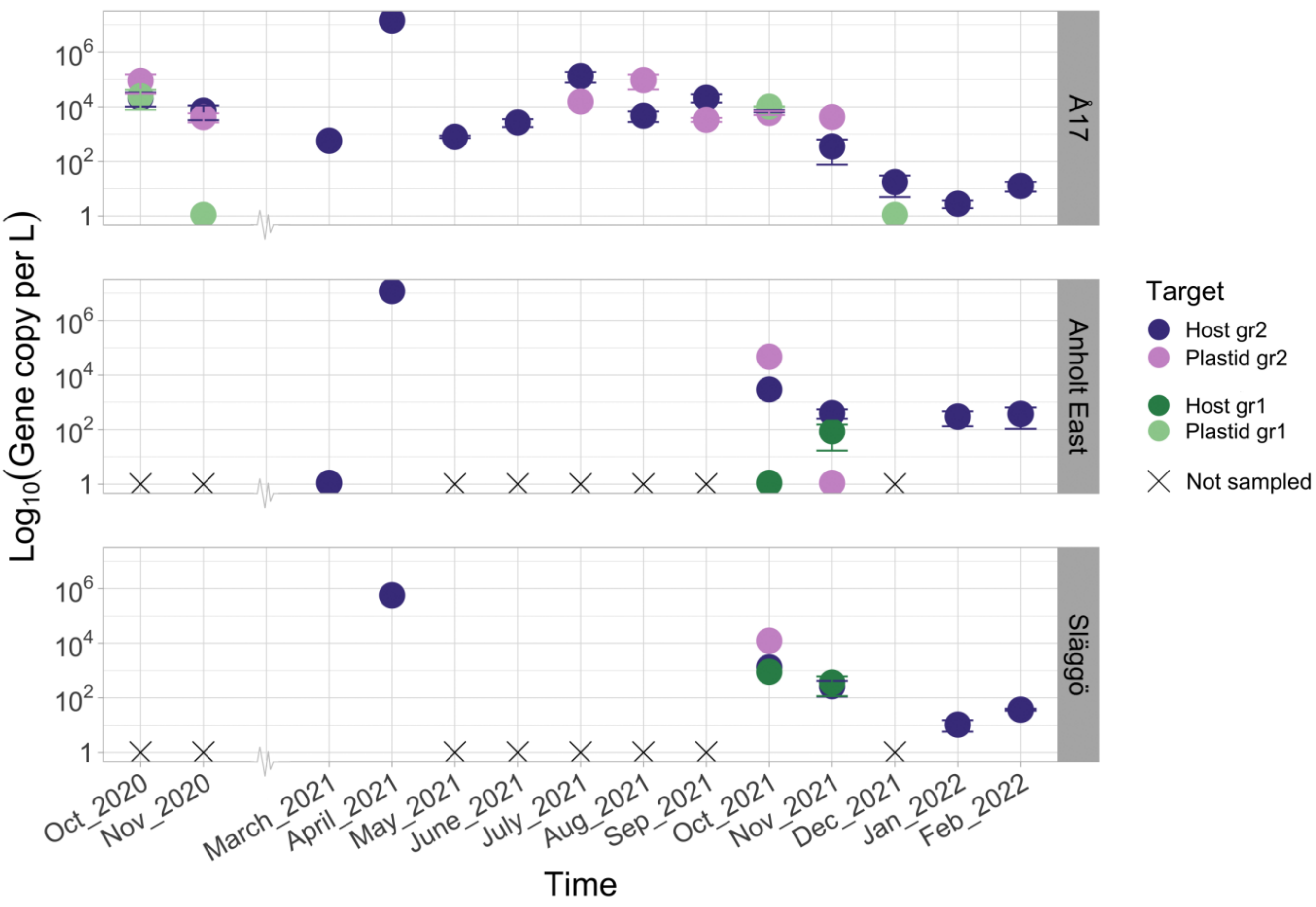

Figure S2. Seasonal dynamics of *Meringosphaera* and its plastid per sampling location, related to Figure 2 and STAR methods. This figure shows the same qPCR abundance data as that in Figure 2A, but here the data has been separated according to sampling location. The upper panel shows the samples from Å17, the middle from Anholt East, and the lowest Släggö. The abundance is reported as the Log of the gene copies L^{-1} and the data are represented as the mean ± SEM. For the host, this refers to the abundance of the *Meringosphaera* group 1 and 2 18S rDNA and for the plastid it refers to the group 1 and group 2 *rbcL* gene. The colour of the point indicates both whether it refers to host or plastid, and the group identity. A cross is shown to indicate when a location was not sampled. The samples were taken monthly between March 2021 and February 2022, in addition two extra samples were taken in October and November 2020. If the target was detected in some but not all of the technical replicates the sample was marked as detected but not quantifiable and were plotted with a value of 1.1

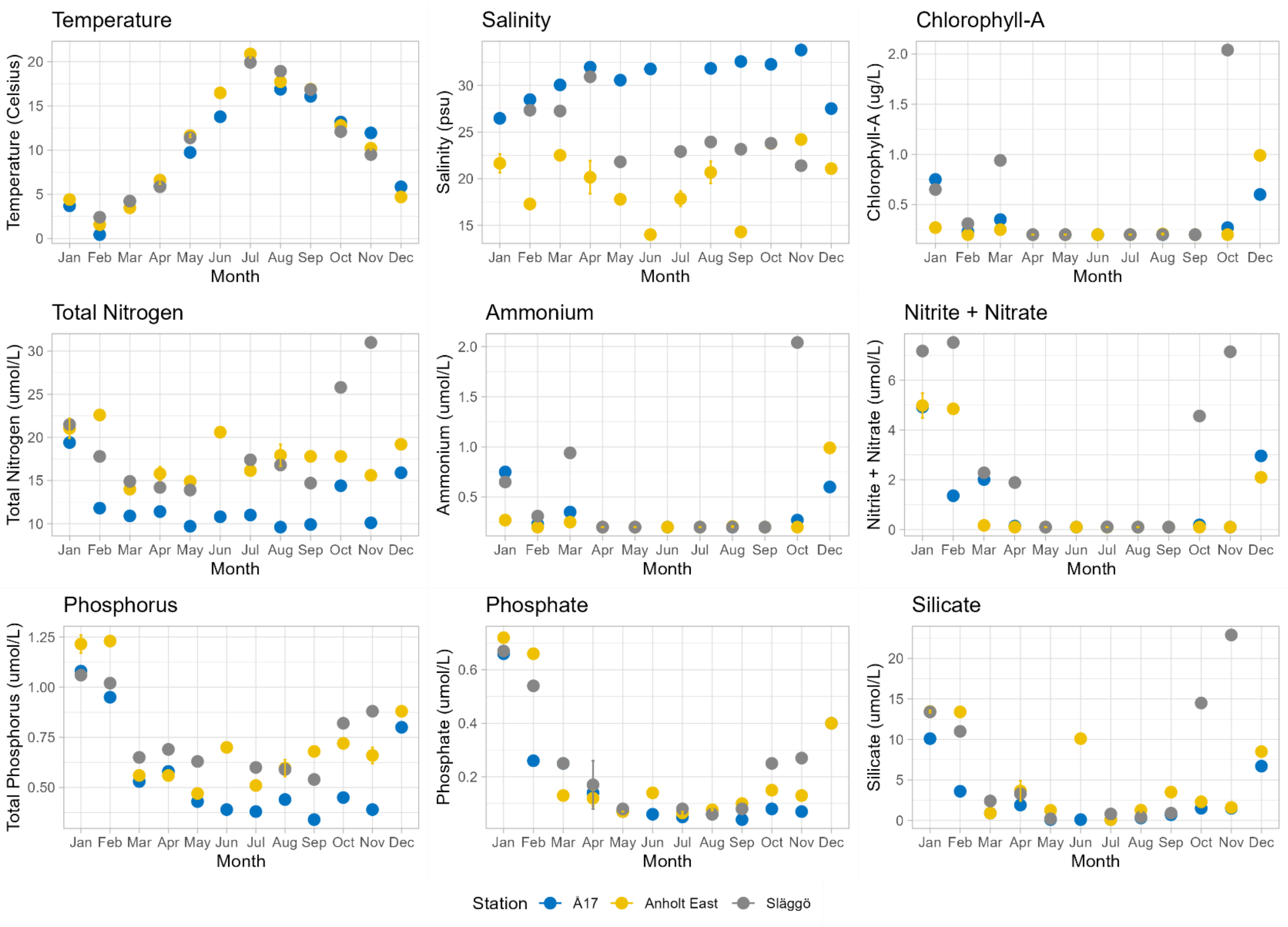

Figure S3. Hydrographic conditions at the sampling locations in 2021, related to Figure 2 and STAR methods. This data was downloaded from the SMHI website (https://sharkweb.smhi.se/hamta-data) to match the locations of the samplings, and to show the hydrographic conditions over the course of a year. The total phosphorus measures all the phosphorus, both soluble and particulate, and the phosphate measures the dissolved inorganic phosphate. The colours represent the three sampling stations: Å17, Anholt East and Släggö. Data are represented as the mean ± SEM.

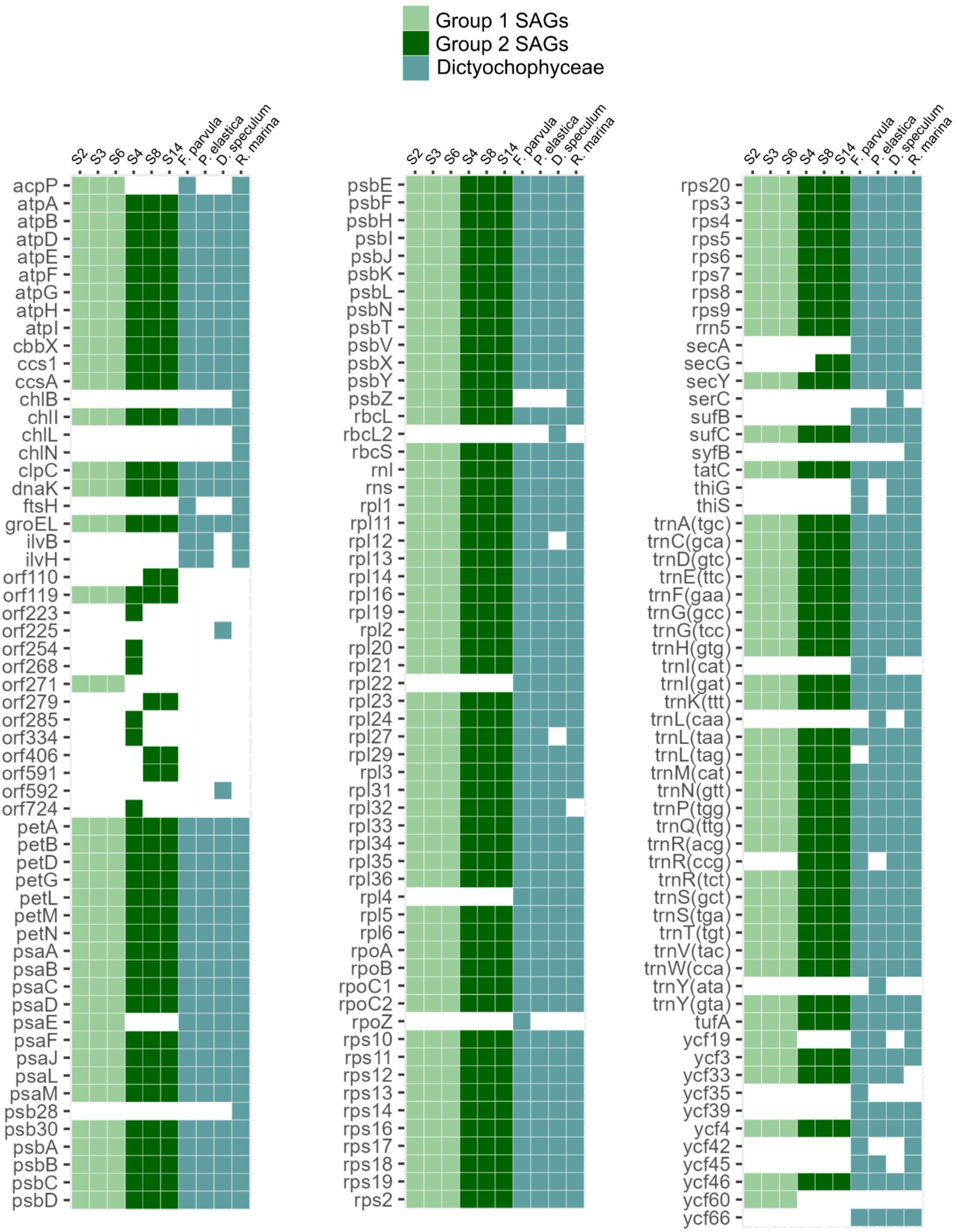

Figure S4. Full comparison of the gene repertoires of the *Meringosphaera* plastid genomes with free-living Dictyochophyceae, related to Figure 4. Compares the presence/absence of annotated genes between the six complete SAG plastids and four free-living photosynthetic Dictyochophyceae. This figure shows the comparison of all annotated genes, while Figure 4A shows only a subset. The background colour of the squares highlights the identity of the plastids (pale green = group 1 SAGs, dark green = group 2 SAGs and pale blue = representative Dictyochophyceae).

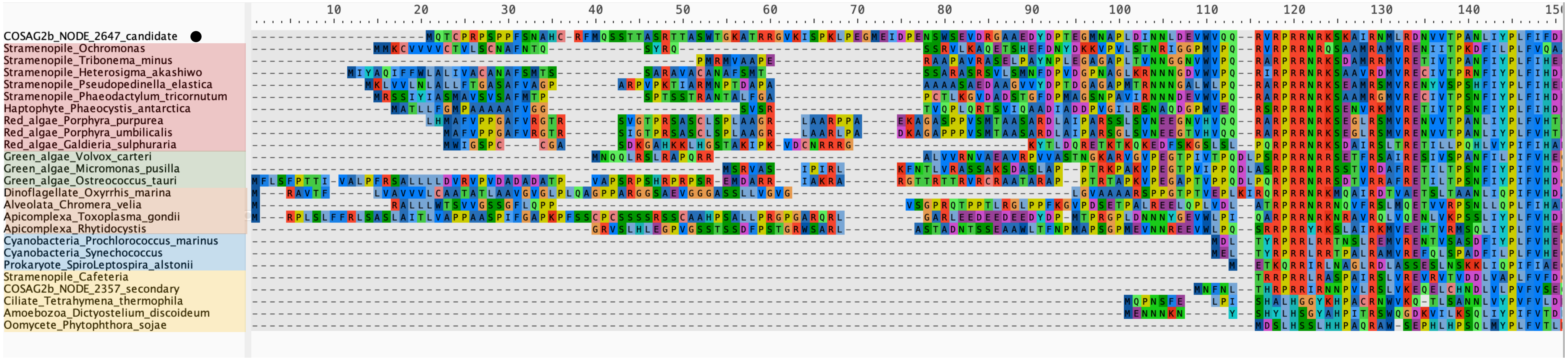

Figure S5. Alignment of the N-terminal extension from a candidate host-encoded plastid-associated protein with predicted plastid-targeting, related to Figure 5. The hemB candidate from COSAG2b (Node 2647) is at the top of the alignment, indicated by the black circle. The first ~150 amino acids are aligned with representatives of different groups: red algae and secondary red plastid-bearing groups (highlighted in red), green algae (highlighted in green), members of the Alveolates (in orange), bacteria (blue), and eukaryotic homologs that are not associated with plastids (yellow). This last group includes node 2357 also from COSAG2b, but this copy is of centrohelid-origin and is predicted to be cytosolic. The alignment was performed by MaffT as described in the Methods section and is visualised here in AliView[S1].

	S1	S2	S3	S4	S5	S6	S7	S8	S9	S10	S11	S12	S13	S14	S15
**Sampling location**	Anholt	Anholt	Anholt	Anholt	Anholt	Anholt	Anholt	Å17	Å17	Å17	Å17	Å17	Å17	Å17	Å17
**Sampling date**	17 Oct 2018	17 Oct 2018	17 Oct 2018	17 Oct 2018	17 Oct 2018	17 Oct 2018	17 Oct 2018	11 Nov 2018	11 Nov 2018	11 Nov 2018	11 Nov 2018	11 Nov 2018	11 Nov 2018	11 Nov 2018	11 Nov 2018
**18S group**	Gr.2	Gr.1	Gr.1	Gr.2	Gr.2	Gr.1	Gr.2	Gr.2	Gr.2	Gr.2	Gr.2	Gr.2	Gr.2	Gr.2	Gr.2
**16S group**	Gr.2	Gr.1	Gr.1	Gr.2		Gr.1		Gr.2				Gr.2		Gr.2	
**rbcL group**	Gr.2	Gr.1	Gr.1	Gr.2		Gr.1	Gr.2	Gr.2			Gr.2	Gr.2		Gr.2	Gr.2
**psbA group**	Gr.2	Gr.1	Gr.1	Gr.2		Gr.1		Gr.2			Gr.2	Gr.2		Gr.2	
**COSAG**	2b	1	1	2b	2b	1	2b	2b	2b	2a	2b	2b	2a	2b	2b
**Plastid complete?**	No	Yes	Yes	Yes		Yes	No	Yes			No	No		Yes	No
**No. of plastid contigs**	1	1	1	1		1	3	1			4	9		1	3
**Total size plastid contig(s) (bp)**	61,040	83,372	83,264	92,424		83,341	3,009	88,018			32,534	72,676		88,018	24,491
**No. of plastid ORFS**		137	137	141		137		139						139	

Table S1: Summary and overview of sample collection and analytical details for the 15 SAGs presented in this work, related to STAR Methods. Here shown are: location and date of the sample collection, the group identity of the host 18S rDNA, and the plastid 16S rDNA, *rbcL* and *psbA* sequences. If no sequence match was found the square is left blank. In SAGs where plastid sequences could be identified, the number of contigs and total size of the plastid sequences, as well as whether the plastid genome is predicted to be complete is included. For the SAGs with a complete plastid genome, the predicted number of ORFs is provided.

Assembly	Accession number	GC content (%)	N50	Assembly size (Mb)	Number of contigs	Fraction of reads that map	BUSCO score incl. plastid	BUSCO score excl. plastid
COSAG 1	-	42.21	16291	120.51	25479	99.95%	-	41.2%
COSAG 2a	-	43.43	9579	109.91	26840	99.92%	-	36.0%
COSAG 2b	-	42.96	14044	422.44	91663	99.96%	-	72.1%
S1	SAMN32532880	44.56	13555	43.66	8894	99.50%	24.3%	23.6%
S2	SAMN32532881	44.69	22578	71.15	11871	99.70%	36.5%	36.5%
S3	SAMN32532882	41.86	21157	45.86	7371	99.67%	31.0%	30.6%
S4	SAMN32532883	45.09	13583	44.70	9505	99.45%	17.6%	17.6%
S5	SAMN32532884	42.71	34128	69.53	8767	99.83%	16.4%	16.4%
S6	SAMN32532885	39.03	24121	39.01	6421	99.83%	13.4%	13.4%
S7	SAMN32532886	44.9	23158	78.77	12828	99.74%	56.1%	55.7%
S8	SAMN32532887	40.8	17863	56.78	10523	99.81%	18.9%	18.5%
S9	SAMN32532888	39.07	17727	57.64	10396	99.78%	17.2%	17.2%
S10	SAMN32532889	42.02	11131	57.00	13094	99.54%	24.7%	24.3%
S11	SAMN32532890	41.45	21939	62.37	9512	99.77%	18.9%	18.9%
S12	SAMN32532891	45.07	14565	61.65	12541	99.69%	21.2%	21.2%
S13	SAMN32532892	45.51	8555	67.30	16956	99.42%	26.7%	26.3%
S14	SAMN32532893	40.44	16806	55.15	9881	99.79%	18.1%	17.7%
S15	SAMN32532894	40.85	17482	44.25	7951	99.77%	14.5%	14.5%

Table S2: Assembly parameters for both the SAGs and COSAGs, related to STAR Methods. The accession numbers are shown for the raw reads that were deposited to the BioSample database under BioProject ID PRJNA917255. The average GC content, N50, number of contigs, and assembly size were calculated with Quast[S2]. Samtools was used to calculate the fraction of the reads that mapped to the assemblies. The BUSCO completeness score was calculated with the eukaryotic database in genome mode. For the SAGs, BUSCO scores have been calculated both including and excluding the plastid contigs in order to facilitate direct comparisons with the COSAGS, which do not contain plastid contigs. S7 is believed to have higher completeness owing to the presence of contamination (as discussed in the methods). The co-assemblies have higher completeness than the majority of the individual SAGs.

Method	Name	Target	HB % Formamide	Temp.	Reference	Component	Sequence
qPCR	Mer18S_831P<br>Mer18S_860F<br>Mer18S_801R	18S group 1	-	60°c	This study	Probe (FAM-TAMRA)<br>Forward primer<br>Reverse primer	CATGGAATACAAATGTCCCCA<br>GTCTTCCATGAATCCAAGAATTTCA<br>CGGACCGACGTAATGATTAATAGG
qPCR	Mer18S_622P<br>Mer18S_602F<br>Mer18S_668R	18S group 2	-	60°c	This study	Probe (FAM-TAMRA)<br>Forward primer<br>Reverse primer	TCATGTGTACGCGAGGTG<br>TGAGCGTCCGTGGCTACTG<br>CGCACGCACTTAGTTAAAAGCA
qPCR	Mer_rbcL_1332P<br>Mer_rbcL_1306F<br>Mer_rbcL_1353R	*rbcL* group 1	-	54°c	This study	Probe (VIC-TAMRA)<br>Forward primer<br>Reverse primer	TCCTGCAATCCTTC<br>GAAGGTCGTGATTACGTAGCAGAA<br>TGAAGAGGACCACACATCTTAGCA
qPCR	Mer_rbcL_1079P<br>Mer_rbcL_1049F<br>Mer_rbcL_1095R	*rbcL* group 2	-	54°c	This study	Probe (VIC-TAMRA)<br>Forward primer<br>Reverse primer	CCTGCCTCAAGGTT<br>CACATTACTAGAAACGCAAACTTCAAT<br>AGCCCAGTCTTGTGCAAAGAA
CARD-FISH	EUK1209R (EUK1195)	General 18S rRNA	30 - 40%	35°C or 46°C	Giovannoni et al., 1988.[S3]	Probe	GGGCATCACAGACCTG
CARD-FISH	Mer482	*Meringosphaera* 18S rRNA	40%	35°C	This study	Probe	CAATGGCTCCTCGATGAT

Table S4: Oligonucleotides used in the qPCR and CARD-FISH assays, related to STAR Methods. The method column denotes if used for qPCR or CARD-FISH. For qPCR, listed are the TaqMAN primers and probes that target both the *Meringosphaera* 18S rDNA group 1 and 2, and the corresponding plastid *rbcL* group 1 and 2. For CARD-FISH, listed are the general eukaryote probe used for the positive control and the probe designed for *Meringosphaera* 18S rRNA. The temperature column shows for the qPCR target sets the Tm and for the CARD-FISH probes the temperature of hybridisation. The optimised formamide concentration in the hybridisation buffer (HB) only applies to the CARD-FISH probes.

Supplemental references

[S1] Larsson, A. (2014). AliView: a fast and lightweight alignment viewer and editor for large datasets. Bioinformatics *30*, 3276–3278. 10.1093/bioinformatics/btu531.

[S2] Gurevich, A., Saveliev, V., Vyahhi, N., and Tesler, G. (2013). QUAST: quality assessment tool for genome assemblies. Bioinformatics *29*, 1072–1075. 10.1093/bioinformatics/btt086.

[S3] Giovannoni, S.J., DeLong, E.F., Olsen, G.J., and Pace, N.R. (1988). Phylogenetic group-specific oligodeoxynucleotide probes for identification of single microbial cells. Journal of Bacteriology *170*, 720–726. 10.1128/jb.170.2.720-726.1988.
